# Supplementary material for: Sex differences in strength at the shoulder: a systematic review
Source: PeerJ. 2024 Mar 20;12:e16968. doi: 10.7717/peerj.16968 (PMC10960529; doi:10.7717/peerj.16968)
Supplement: Supplemental Information 2 [file peerj-12-16968-s002.docx]

# **Supplementary Table 1: Extracted anthropometric and methodology data.**

| Title | Participant Anthropometrics | Movement Direction | Movement Type | Measurement Method | Measurement Unit |
| --- | --- | --- | --- | --- | --- |
| Kim, et al., 2009 | N: 107 (M); 76 (F)  Age: 59.6±11.3  Height: 1.73±11.2  Weight: 83±17.9 | Abduction;  External Rotation | Isometric | Isobex Dynamometer | Nm |
| Huberman, et al., 2020 | N: 30 (M); 157 (F); 2 (nonidentified)  Age: 31.9±3.5 | Flexion;  Extension;  Abduction;  Adduction;  Horizontal Flexion;  Horizontal Extension;  Internal Rotation;  External Rotation | Isometric | Digital Hand-Held Dynamometer | Ibs |
| Murray, et al., 1985 | N: 20 (M); 20 (F)  Male:  Young Age: 31  Height: 1.79  Weight: 74  Older Age: 62  Height: 1.77  Weight: 85  Female:  Young Age: 29  Height: 1.62  Weight: 56  Older Age: 62  Height: 1.60  Weight: 71 | Flexion;  Extension;  Abduction;  Adduction;  Internal Rotation;  External Rotation | Isometric | Deflection-Beam Force Gauges | kg-cm |
| Roy, et al., 2009 | N: 121 (M); 173 (F)  Male:  Young Age: 27.1±5.5  Mid Age: 48.5±5.3  Older Age: 68.2±5.7  Female:  Young Age: 25.4±6.2  Mid Age: 48.8±5.1  Older Age: 64.9±4.8 | Internal Rotation;  External Rotation | Isometric | Computerized Dynamometer | Nm |
| Chezar, et al., 2013 | N: 200 (M); 200 (F)  Male:  Age: 30-39; n = 50  Age: 40-49; n = 50  Age: 50-59; n = 50  Age: 60-69; n = 50  Female:  Age: 30-39; n = 50  Age: 40-49; n = 50  Age: 50-59; n = 50  Age: 60-69; n = 50 | Abduction;  Internal Rotation;  External Rotation | Isometric | Dynamometer | Nm/kg |
| Yates, et al., 1980 | N: 9 (M); 9 (F)  Male:  Age: 25.2±2.7  Height: 1.80±0.05  Weight: 73±7.6  Female:  Age: 28.3±6.1  Height: 1.64±0.07  Weight: 56.9±8.6 | Flexion | Isometric | Stoelting Grip Dynamometer | N |
| Lannersten, et al., 1993 | N: 179 (M); 179 (F)  Male:  Age: 41.7±13.3  Height: 1.79±0.07  Weight: 79±15.2  Female:  Age: 42.7±12.9  Height: 1.65±0.06  Weight: 65±10.4 | Flexion;  Abduction;  External Rotation | Isometric | Electromechanical Force Transducer | Nm |
| Gil Coury, et al., 1996 | N: 15 (M); 15 (F)  Male:  Age: 22.27 ± 2.89  Height: 1.77 ± 0.10  Weight: 72.33 ± 11.37  Female:  Age: 22.20 ± 1.97  Height: 1.67 ± 0.75  Weight: 58.80 ± 7.07 | Adduction | Isometric | Load Cell (MM type) | N |
| Barnekow-Bergkvist, et al., 2007 | N: 48 (M); 17 (F)  Male:  Age: 35.7 ± 8.8  Height: 1.80 ± 0.06  Weight: 84.4 ± 12.3  Female:  Age: 31.9 ± 7.7  Height: 1.68 ± 0.07  Weight: 69.2 ± 7.3) | Flexion | Isometric | Strain Gauge Dynamometer, Biodex Isokinetic Dynamometer | N, Nm |
| MacDonell and Keir, 2005 | N: 8 (M); 7 (F)  Male:  Age: 30.4±3.8  Height: 1.79±0.06  Weight: 79.9±5.2)  Female:  Age: 29.6±3.8  Height: 1.62±0.08  Weight: 58.2±4.9 | Flexion;  Abduction | Isometric | Cybex II Dynamometer | Nm |
| Faber, et al., 2006 | N: 213 (M); 210 (F)  Male:  Height: 1.78  Weight: 82  Female:  Height: 1.66  Weight: 67 | Flexion;  Abduction | Isometric | Dynamometer | Nm |
| Meldrum, et al., 2007 | N: 235 (M); 259 (F)  Male:  Age: 44.3±15.2  Height: 1.76±0.08  Weight: 82.5±11.7  Female:  Age: 44.14±14.6  Height: 1.62±0.06  Weight: 66.3±11.19 | Abduction;  Adduction | Isometric | Quantitative Muscle Assessment System | Kg |
| Riemann, et al., 2010 | N: 90 (M); 91 (F)  Male:  Age: 23.3±4.8  Height: 1.79±0.08  Weight: 81.4±14.0  Female:  Age: 23.4±4.1  Height: 1.64±0.08  Weight: 64.7±16.9 | Internal Rotation;  External Rotation | Isometric | Hand-Held Baseline 250 Hydraulic Push-Pull Dynamometer | Percent Body Mass |
| VanHarlinger, et al., 2015 | N: 90 (M); 90 (F) | Flexion;  Extension;  Abduction;  Horizontal Flexion;  Horizontal Extension;  Internal Rotation;  External Rotation | Isometric | Nicholas Manual Muscle Tester | Kg |
| McKay, et al., 2017 | N: 350 (M); 350 (F) | Internal Rotation;  External Rotation | Isometric | Citec Hand-Held Dynamometer | N |
| Collins and O'Sullivan, 2018 | N: 20 (M); 20 (F)  Male:  Young Age: 26.0± 2.18  Old Age: 59.6±3.17  Female:  Young Age: 26.0± 2.18  Old Age: 59.6±3.17 | Abduction | Isometric | Commercial Force Meter | N |
| Harbin, et al., 2020 | N: 69065 (M); 38037 (F)  Male:  AR: 20-29  Height: 1.79 ± 0.08  Weight: 88.1 ± 20.6  N = 28212  AR: 30-39  Height: 1.78 ± 0.08  Weight: 93.0 ± 21.0  N = 16217  AR: 40-49  Height: 1.80 ± 0.08  Weight: 93.4 ± 20.5  N = 11212  AR: 50-59  Height: 1.78 ± 0.08  Weight: 93.2 ± 19.7  N = 5863  AR: 60-69  Height: 1.77 ± 0.21  Weight: 93.7 ± 18.8  N = 1068  Female:  AR: 20-29  Height: 1.65 ± 0.07  Weight: 74.9 ± 20.5  N = 15257  AR: 30-39  Height: 1.65 ± 0.08  Weight: 80.1 ± 21.5  N = 8457  AR: 40-49  Height: 1.64 ± 0.07  Weight: 79.3 ± 20.5  N = 5917  AR: 50-59  Height: 1.64 ± 0.07  Weight: 78.9 ± 19.0  N = 3286  AR: 60-69  Height: 1.62 ± 0.06  Weight: 78.5 ± 17.4  N = 675 | Flexion | Isometric | Testing equipment specially made by Occupational Performance Corporation | kg |
| Lorenzo and Nunez, 2021 | N: 78 (M); 78 (F)  Male:  Age: 24±1  Female:  Age: 24±0.8 | Abduction | Isometric | Handheld IDO Isometer | Kg |
| Ferreira, et al., 2020 | N: 7 (M); 7 (F)  Male:  Age: 24.71 ± 3.73  Height: 1.84 ± 0.7  Weight: 77.27 ± 4.72  Female:  Age: 22.71 ± 4.23  Height: 1.64 ± 0.7  Weight: 59.84 ± 6.68 | Flexion;  Extension | Isometric | Torque Meter (SMS1) | Nm/kg |
| Hills and Bohannon, 1992 | N: 14 (M); 16 (F)  Age: 31 (20-48)  Weight: 641 N (418- 1224 N) | Extension | Isometric | Ametek Accuforce II Hand-Held Dynamometer | N |
| Holzbaur, et al., 2007 | N: 5 (M); 5 (F)  Male:  Age: 29.2±4.4  Height: 1.78±0.06  Weight: 81.6±8.9  Female:  Age: 28.0±5.1  Height: 1.65±0.08  Weight: 56.7±9.6 | Abduction;  Adduction | Isometric | Biodex System3 | Nm |
| Westrick, et al., 2013 | N: 546 (M); 73 (F)  Male:  Age: 18.8±1  Height: 1.79±7.5  Weight: 75.3±12.2  Female:  Age: 18.7±0.9  Height: 1.65±7  Weight: 62.6±7 | Flexion;  Abduction;  Internal Rotation;  External Rotation | Isometric | Handheld Dynamometer | N/kg |
| Hughes, et al., 1999 | N: 60 (M); 60 (F)  Age: 44±15  Height: 1.72±0.10  Weight: 76±14 | Flexion;  Extension;  Abduction;  Adduction;  Internal Rotation; External Rotation | Isometric | Cybex II Isokinetic Dynamometer | Nm |
| Magnusson, et al., 1995 | N: 13 (M); 11 (F)  Male:  Age: 31.2±1.3  Height: 1.78±1.3  Weight: 78.9±2.8  Female:  Age: 29.4±1.3  Height: 1.65±2.3  Weight: 63.2±2.4 | Abduction;  Internal Rotation;  External Rotation | Isometric | Hand-Held Dynamometer | Nm/kg |
| Stausholm, et al., 2021 | N: 19 (M); 10 (F)  Male:  Age: 26.2±4.6  Weight: 78.4±4.9  Female:  Age: 25±2.9  Weight: 65±6.3 | Internal Rotation;  External Rotation | Isometric | Hand-Held Dynamometer | Nm/kg |
| Guirelli, et al., 2021 | N: 24 (M); 25 (F)  Male:  Age: 22.7±3.2  Height: 1.76 ± 0.7  Weight: 74.6±8.1  Female:  Age: 23±2.7  Height: 1.63 ± 0.56  Weight: 61.5±9.1 | Abduction;  External Rotation | Isometric | Handheld Dynamometer | N/kg |
| Douma, et al., 2014 | N: 259 (M); 203 (F)  Male:  Age: 25.2 ± 3  Height: 1.82 ± 0.8  Weight: 74.3 ± 10  Age: 33.6 ± 3  Height: 1.81 ± 0.8  Weight: 80.6 ± 13  Age: 44.9 ± 3  Height: 1.83 ± 0.8  Weight: 82.4 ± 10  Age: 54.1 ± 3  Height: 1.81 ± 0.7  Weight: 82.4 ± 14  Female:  Age: 25.9 ± 3  Height: 1.72 ± 0.6  Weight: 68.1 ± 13  Age: 34.8 ± 3  Height: 1.71 ± 0.8  Weight: 68.2 ± 9  Age: 44.3 ± 3  Height: 1.70 ± 0.7  Weight: 68.2 ± 9  Age: 53.6 ± 3  Height: 1.67 ± 0.12  Weight: 67.8 ± 12 | Abduction | Isometric | Hand-held Dynamometer | Nm |
| Pontillo and Sennet, 2020 | N: 270 (M); 206 (F)  Age: 18 | Flexion;  Internal Rotation;  External Rotation | Isometric | Hand-held Dynamometer | kg |
| Andrews, et al., 1996 | N: 51 (M); 54 (F)  Male:  Younger Age: 54±3.4  Height: 1.76±0.07  Weight: 835.7±101.3N  Older Age: 66.1±2.9  Height: 1.76±6.1  Weight: 771.3±105N  Female:  Younger Age: 54.6±2.8  Height: 1.63±0.07  Weight: 684.9±143.4N  Older Age: 64.5±2.9  Height: 1.61±6.2  Weight: 645.2±79N | Flexion;  Extension;  Abduction;  Internal Rotation;  External Rotation | Isometric | Chatillon CSD400C hand-held dynamometer | N |
| Busko & Gajewski, 2011 | N: 8 (M); 8 (F)  Male:  Age: 21.9 ± 3.2  Height: 1.88 ± 0.6  Weight: 82.5 ± 3.8  Female:  Age: 19.4 ± 3.7  Height: 1.75 ± 0.09  Weight: 66.4 ± 8.9 | Flexion;  Extension | Isometric | Dynamometer | Nm |
| Alizadehkhaiyat, et al., 2014 | N: 22 (M); 23 (F)  Male:  Age: 30.8 ± 8.2  Height: 1.77 ± 0.05  Weight: 81.1 ± 13.7  Female:  Age: 29.7 ± 10.4  Height: 1.65 ± 0.06  Weight: 62.6 ± 9.7 | Flexion;  Abduction;  Internal Rotation;  External Rotation | Isometric | Nottingham Mecmesin Myometer | N |
| Eren, et al., 2019 | N: 34 (M); 46 (F)  Age: 23.8 (18-30)  Male:  Weight: 74.9±9.5  Female:  Weight: 59.9±11.3 | Scaption | Isometric | Electronic Dynamometer | N |
| Backman, et al., 1995 | N: 55 (M); 53 (F)  Male:  Age: 24.4 ± 3.3  Height: 1.76 ± 0.07  Weight: 71 ± 9  n = 12  Age: 35.1 ± 2.6  Height: 1.82 ± 0.08  Weight: 82 ± 11  n = 11  Age: 43.8 ± 3.5  Height: 1.80 ± 0.06  Weight: 74 ± 9  n = 10  Age: 54.0 ± 3.3  Height: 1.80 ± 0.06  Weight: 76 ± 5  n = 10  Age: 65.6 ± 3.1  Height: 1.78 ± 0.04  Weight: 75 ± 7  n = 12  Female:  Age: 23.4 ± 2.7  Height: 1.70 ± 0.09  Weight: 61 ± 8  n = 10  Age: 34.8 ± 3.0  Height: 1.68 ± 0.07  Weight: 63 ± 6  n = 10  Age: 43.1 ± 2.8  Height: 1.66 ± 0.06  Weight: 63 ± 11  n = 13  Age: 54.0 ± 2.9  Height: 1.68 ± 0.07  Weight: 64 ± 8  n = 10  Age: 65.4 ± 2.8  Height: 1.63 ± 0.08  Weight: 65 ± 2  n = 10 | Abduction | Isometric | Dynamometer | N |
| Cools, et al., 2016 | N: 101 (M); 100 (F)  Male:  Age: 27.3± 8.3  Height: 1.83 ± 0.06  Weight: 80.4 ± 11.2  Female:  Age: 27.8 ± 8.5  Height: 1.69 ± 0.07 Weight: 65.8 ± 9.3 | Internal Rotation;  External Rotation | Isometric | Hand-Held Dynamometer | N |
| Kramer and Ng, 1995 | N: 20 (M); 20 (F)  Male:  Age: 58±9  Height: 1.76±0.04  Weight: 856±140N  Female:  Age: 59±9  Height: 1.62±0.05  Weight: 650±104N | Internal Rotation;  External Rotation | Isometric | Isokinetic Dynamometer and Hand-Held Dynamometer | Nm |
| Marcondes, et al., 2019 | N: 10 (M); 10 (F)  Male:  Age: 26±4  Weight: 82.7±12.5  Female:  Age: 21±2  Weight: 70.2±15.1 | Flexion;  Extension;  Abduction;  Adduction;  Internal Rotation;  External Rotation | Isokinetic;  60 °/s;  180 °/s | Isokinetic Dynamometer | Percent Body Mass |
| Cahalan, et al., 1989 | N: 26 (M); 24 (F)  Male:  Age: 28.85 ± 6.25  Height: 1.79 ± 0.07  Weight: 81.15 ± 9.70  Female:  Age: 29.04 ± 5.92  Height: 1.66 ± 0.07  Weight: 62.73 ± 10.91 | Flexion;  Extension;  Abduction;  Adduction;  Internal Rotation;  External Rotation | Isokinetic;  60 °/s; 180 °/s; 300 °/s | Cybex II Isokinetic Dynamometer | N, Nm |
| Shklar and Dvir, 1995 | N: 15 (M); 15 (F)  Age Range: 22-35 | Flexion;  Extension;  Abduction;  Adduction;  Internal Rotation;  External Rotation | Isokinetic;  60 °/s; 120 °/s; 180 °/s | KINCOM II Isokinetic Dynamometer | Nm |
| Koski and McGill, 1994 | N: 26 (M); 25 (F)  Male:  Age: 22  Height: 1.65±0.05  Weight: 65.3±10.5  Female:  Age: 22.5  Height: 1.77±0.07  Weight: 80.0±13.2 | Flexion | Isokinetic;  50 °/s | KIN-COM | Nm |
| Ivey, et al., 1985 | N: 18 (M); 13 (F)  Age: 27 (21-50)  Weight: 152.6 | Flexion;  Extension;  Abduction;  Adduction;  Internal Rotation;  External Rotation | Isokinetic;  60 °/s; 180 °/s | Cybex II | Foot-Pounds |
| Reid, et al., 1989 | N: 20 (M); 20 (F)  Male:  Age: 25  Female:  Age: 27 | Abduction;  Adduction;  Internal Rotation;  External Rotation | Isokinetic;  60 °/s | Cybex II Isokinetic Dynamometer | Nm |
| McMaster, et al., 1992 | N: 24 (M); 23 (F)  Male:  Age: 22  Height: 1.81  Weight: 80  Female:  Age: 23  Height: 1.72  Weight: 64 | Abduction;  Adduction;  Internal Rotation;  External Rotation | Isokinetic;  30 °/s; 180 °/s | Cybex II Isokinetic Dynamometer | Foot-Pounds |
| Sanchez, et al., 1999 | N: 30 (M); 30 (F)  Male:  Age: 19.5 ± 1.59  Height: 1.76 ± 0.62  Weight: 76.88 ± 17.59  Female:  Age: 19.23 ± 1.10  Height: 1.62 ± 0.07  Weight: 56.95 ± 7.92 | Abduction;  Adduction | Isokinetic;  60 °/s; 120 °/s | Biodex 2000 Multi System Joint Dynamometer | Nm |
| Sanchez, et al. 2000 | N: 20 (M); 20 (F)  Male:  Age: 19.54± 1.70  Height: 1.76 ± 0.61  Weight: 75.43 ± 10.90  Female:  Age: 19.45 ± 1.10  Height: 1.62 ± 0.07  Weight: 57.48 ± 7.10 | Abduction;  Adduction | Isokinetic;  60 °/s; 120 °/s | Electromagnetic Dynamometer | Nm |
| Khalaf and Parnianpour, 2001 | N: 10 (M); 10 (F)  Male:  Age: 26.2±3.8  Height: 1.79±10.7  Weight: 85.1±14  Female:  Age: 24.2±2.6  Height: 1.65±0.08  Weight: 58.3±7.2 | Flexion;  Extension | Isokinetic;  10 °/s, 50 °/s, 100 °/s, 150 °/s, 200 °/s, 250 °/s | KIN-COM 125E | Nm |
| Motta, et al., 2019 | N: 30 (M); 30 (F)  Male:  Age: 30±4.4  Height: 1.75±0.1  Weight: 81.4±8.9  Female:  Age: 29.8±5.2  Height: 16.4±0.1  Weight: 63.1±8.2 | Internal Rotation;  External Rotation | Isokinetic;  60 °/s; 240 °/s | Isokinetic Dynamometer | Nm/kg |
| Maddux, et al., 1989 | N: 19 (M); 19 (F)  Male:  Age: 34±10  Weight: 184±41  Female:  Age: 26±6  Weight: 135±22 | Internal Rotation;  External Rotation | Isokinetic;  60 °/s;  180 °/s | Cybex Dynamometer | Foot-Pounds |
| Hartsell, 1998 | N: 11 (M); 11 (F)  Male:  Age: 27.2±2.6  Height: 1.82±0.06  Weight: 84.3±11.4  Female:  Age: 25.4±3.1  Height: 1.66±0.08  Weight: 65.1±9.38 | Internal Rotation;  External Rotation | Isokinetic;  60 °/s;  120 °/s;  180 °/s | Cybex 770 Isokinetic Dynamometer | Nm |
| VanMeeteren, et al., 2002 | N: 10 (M); 10 (F)  Age: 27±9.6  Height: 1.77  Weight: 70 | Abduction;  Adduction;  Internal Rotation;  External Rotation | Isokinetic;  60 °/s;  120 °/s;  180 °/s | Biodex Dynamometer | Nm |
| Hill, et al., 2005 | N: 11 (M); 6 (F) | Internal Rotation;  External Rotation | Isokinetic;  60 °/s;  90 °/s  120 °/s; | Cybex 770 Norm Isokinetic System | Nm |
| Hageman, et al., 1989 | N: 9 (M); 10 (F)  Age: 21-33 | Internal Rotation;  External Rotation | Isokinetic;  60 °/s;  180 °/s | KIN-COM Robotic Dynamometer | Nm |
| Nyberg, et al., 2014 | N: 15 (M); 15 (F)  Male:  Age: 61.4±7.3  Height: 1.80±6.5  Weight: 82.2±12.1  Female:  Age: 58.9±6.5  Height: 1.67±6.4  Weight: 64.3±6.5 | Flexion | Isokinetic;  60 °/s | KINCOM Isokinetic Dynamometer | N |
| VanCingel, et al., 2007 | N: 9 (M); 7 (F)  Male:  Age: 25.4±5.2  Height: 1.84±6.9  Weight: 78.1±8.8  Female:  Age: 21.6±5.4  Height: 1.73±7.1  Weight: 66.9±6.2 | Internal Rotation;  External Rotation | Isokinetic;  60 °/s;  120 °/s | CYBEX 6000 Dynamometer | Nm/kg |
| Murgia, et al., 2018 | N: 10 (M); 10 (F)  Young Age: 23.8±1.54  Height: 1.79±0.11  Weight: 75.67+14.15  Old Age: 60.9±8.53  Height: 1.69±0.07  Weight: 87.65±20.6 | Flexion;  Abduction;  Internal Rotation;  External Rotation | Isokinetic;  60 °/s;  90 °/s | KINCOM Dynamometer | Nm |
| Barrenetxea-Garcia, et al., 2019 | N: 17 (M); 10 (F)  Male:  Age: 23.67 ± 6.16  Height: 1.79 ± 0.07  Weight: 81.67 ± 9.36  Female:  Age: 24.17 ± 5.07  Height: 1.65 ± 0.08  Weight: 58.5 ± 7.58 | Internal Rotation;  External Rotation | Isokinetic;  60 °/s;  240 °/s | Isokinetic Machine (Humac Norm, USA) | Nm |
| Ellenbecker and Roetert, 2003 | N: 31 (M); 35 (F)  Age: 18-21 | Internal Rotation;  External Rotation | Isokinetic;  210 °/s;  300 °/s | Isokinetic Dynamometer | Nm/kg |
| Mayer, et al., 1994 | N: 32 (M); 19 (F)  Male:  Age: 25.7±3.8  Height: 1.81±0.05  Weight: 72.1±2.6  Female:  Age: 24.2±4.7  Height: 1.73±0.05  Weight: 60.8±6.8 | Flexion;  Extension;  Abduction;  Adduction;  Internal Rotation;  External Rotation | Isometric; Isokinetic;  300 °/s; 240 °/s; 180 °/s; 60 °/s;  -60 °/s;  -120 °/s;  -180 °/s;  -240 °/s | Isokinetic Strength Measuring System Lido Active | Nm |
| Smith, et al., 2001 | N: 5 (M); 5 (F)  Age: 27.7±6.39  Height: 1.73±0.09  Weight: 74.4±17.4 | Internal Rotation;  External Rotation | Isometric; Isokinetic;  90 °/s | KINCOM Robotic Dynamometer | Nm |
| Lindstrom, et al., 2003 | N: 13 (M); 14 (F)  Male:  Age: 38±7  Height: 1.84±0.07  Weight: 78±10  Female:  Age: 35±8  Height: 1.68±0.05  Weight: 63±8 | Flexion | Isometric; Isokinetic;  30 °/s;  90 °/s | Isokinetic Dynamometer | N |
| Aydin, et al., 2000 | N: 35 (M); 33 (F)  Male:  Age: 23.5 ± 3.4  Height: 1.79 ± 0.65  Weight: 75.4 ± 8.8  Female:  Age: 20.7 ± 4.2  Height: 1.65 ± 0.05  Weight: 57.9 ± 6.0 | Internal Rotation | Isometric; Isokinetic;  90 °/s | Cybex NORM, Dynamometer | Nm, W |
| Danneskiold-Samsoe, et al., 2009 | N: 53 (M); 121 (F)  Male:  Age: 27.9± 1.2  Height: 1.77 ± 0.07  Weight: 73.8 ± 8.4  Age: 34.3± 2.5  Height: 1.77 ± 0.05  Weight: 77.6 ± 6.8  Age: 43.8± 2.7  Height: 1.78 ± 0.06  Weight: 79.9 ± 10.2  Age: 54.6± 2.0  Height: 1.79 ± 0.07  Weight: 85.8 ± 12.8  Age: 64.1± 2.2  Height: 1.71 ± 0.05  Weight: 76.4 ± 8.5  Age: 74.8 ± 2.8  Height: 1.72 ± 0.05  Weight: 78.3 ± 9.1  Female:  Age: 26.1± 2.2  Height: 1.68 ± 0.05  Weight: 62.8 ± 12.2  Age: 34.7± 2.8  Height: 1.68 ± 0.06  Weight: 65.9 ± 10.5  Age: 44.4± 3.1  Height: 1.67 ± 0.06  Weight: 69.5 ± 11.5  Age: 55.1± 2.9  Height: 1.60 ± 0.06  Weight: 69.6 ± 9.8  Age: 64.5 ± 3.0  Height: 1.80 ± 0.06  Weight: 68.7 ± 13.9  Age: 73.1 ± 2.4  Height: 1.59 ± 0.06  Weight: 65.4 ± 9.5 | Flexion;  Extension;  Abduction;  Adduction | Isometric; Isokinetic;  30 °/s; 60 °/s; 90 °/s; 120 °/s | Isokinetic Dynamometer | N, Nm |
| Kramer and Ng, 1996 | N: 20 (M); 20 (F)  Male:  Age: 57.9±8.8  Height: 1.76±0.04  Weight: 856±140N  Female:  Age: 59.1±9.3  Height: 1.62±0.05  Weight: 650±0.05N | Internal Rotation;  External Rotation | Isometric; Isokinetic;  0 °/s;  60 °/s;  120 °/s | Computerized Dynamometer | Nm |
| Harbo, et al., 2012 | N: 93 (M); 85 (F)  Male:  Age: 24±5  Height: 1.79±0.06  Weight: 74±8  Age: 34±4  Height: 1.80±0.05  Weight: 82±12  Age: 45±3  Height: 1.79±0.04  Weight: 82±9  Age: 55±3  Height: 1.81±0.05  Weight: 82±13  Age: 64±2  Height: 1.79±0.05  Weight: 81±11  Female:  Age: 25±4  Height: 1.68±0.09  Weight: 59±10  Age: 35±3  Height: 1.69±0.07  Weight: 64±6  Age: 44±3  Height: 1.67±0.06  Weight: 61±7  Age: 56±2  Height: 1.67±0.07  Weight: 64±11  Age: 63±3  Height: 1.63±0.05  Weight: 63±12 | Abduction;  Adduction | Isometric;  Isokinetic;  60 °/s | Biodex System 3 PRO Dynamometer | Nm |
