# Supplementary material for: Sex differences in strength at the shoulder: a systematic review
Source: PeerJ. 2024 Mar 20;12:e16968. doi: 10.7717/peerj.16968 (PMC10960529; doi:10.7717/peerj.16968)
Supplement: Supplemental Information 3 — Isometric (ISO) and isokinetic (IKO) data of concentric (Con) and Eccentric (Ecc) movement types. Age ranges (AR) included. Outcomes are relative to the described measurement unit; where available, effect sizes were extracted or calculated (Cohen’s d). [file peerj-12-16968-s003.docx]

# **Supplementary Table 2: Extracted data for studies with shoulder abduction data.**

Isometric (ISO) and isokinetic (IKO) data of concentric (Con) and Eccentric (Ecc) movement types. Age ranges (AR) included. Outcomes are relative to the described measurement unit; where available, effect sizes were extracted or calculated (Cohen's d).

| **Title** | **Movement Type** | **Measurement Unit** | **Outcomes** | **Effect Size (Cohen’s d)** |
| --- | --- | --- | --- | --- |
| Kim, et al., 2009 | Isometric | Nm | Males:  AR: 40-49 = 102.0±27.5  AR: 50-59 = 97.1±16.7  AR: 60-69 = 85.3±18.6  Females:  AR: 40-49 = 54.9±10.8  AR: 50-59 = 56.9±12.8  AR: 60-69 = 49.0±10.8 | AR: 40-49 = 1.71  AR: 50-59 = 2.41  AR: 60-69 = 1.95 |
| Murray, et al., 1985 | Isometric | kg-cm | Males:  Young 45° = 562±23  Old 45° = 426±21  Females:  Young 45° = 275±15  Old 45° = 222±16 | Young 45° = 12.48  Old 45° = 9.71 |
| Chezar, et al., 2013 | Isometric | Nm/kg | Males:  AR: 30-39 = 102±35  AR: 40-49 = 110±35  AR: 50-59 = 122±43  AR: 60-69 = 89±35  Females:  AR: 30-39 = 53±17  AR: 40-49 = 63±23  AR: 50-59 = 57±25  AR: 60-69 = 53±17 | AR: 30-39 = 1.4  AR: 40-49 = 1.34  AR: 50-59 = 1.52  AR: 60-69 = 1.03 |
| Lannersten, et al., 1993 | Isometric | Nm | Males:  AR: 19-34 = 71.6±27.9  AR: 35-44 = 70.3±27.9  AR: 45-65 = 63.6±20.3  Females:  AR: 19-34 = 31.1±10.9  AR: 35-44 = 29.9±10.2  AR: 45-65 = 27.2±8.2 | AR: 19-34 = 1.45  AR: 35-44 = 1.45  AR: 45-65 = 1.79 |
| Barnekow-Bergkvist, et al., 2007 | Isometric | N, Nm | Males:  1420.0±220.0  Females:  940.0±210.0 | 1.52 |
| MacDonell and Keir, 2005 | Isometric | Nm | Males:  30° = 75.7±10  60° = 67.5±7.4  90° = 69.8±7.1  Females:  30° = 41.9±4  60° = 44.5±3  90° = 39.6±3 | 30° = 3.38  60° = 3.11  90° = 4.25 |
| Faber, et al., 2006 | Isometric | Nm | Males:  Young = 71  Middle Age = 73  Elderly = 69  Females:  Young = 32  Middle Age = 31  Elderly = 31 | N/A |
| Meldrum, et al., 2007 | Isometric | Kg | Males:  AR: 20 (Right) = 19.9±12.8  AR: 20 (Left) = 18.6±10.1  AR: 25 (Right) = 20.2±11.9  AR: 25 (Left) = 19±10.1  AR: 30 (Right) = 20±11.4  AR: 30 (Left) = 18.8±10.1  AR: 35 (Right) = 19.6±11  AR: 35 (Left) = 18.4±10.1  AR: 40 (Right) = 19±10.6  AR: 40 (Left) = 17.8±10.1  AR: 45 (Right) = 18.4±10.2  AR: 45 (Left) = 17.1±10.1  AR: 50 (Right) = 17.7±9.8  AR: 50 (Left) = 16.5±10.1  AR: 55 (Right) = 17±9.4  AR: 55 (Left) = 15.7±10.1  AR: 60 (Right) = 16.2±8.9  AR: 60 (Left) = 15±10.1  AR: 65 (Right) = 15.5±8.3  AR: 65 (Left) = 14.2±10.1  Females:  AR: 20 (Right) = 11.2±7.8  AR: 20 (Left) = 10±7.1  AR: 25 (Right) = 11.6±6.9  AR: 25 (Left) = 10.3±6.3  AR: 30 (Right) = 11.4±6.4  AR: 30 (Left) = 10.1±5.7  AR: 35 (Right) = 10.9±5.9  AR: 35 (Left) = 9.7±5.3  AR: 40 (Right) = 10.4±5.6  AR: 40 (Left) = 9.1±4.9  AR: 45 (Right) = 9.7±5.2  AR: 45 (Left) = 8.5±4.6  AR: 50 (Right) = 9±4.8  AR: 50 (Left) = 7.8±4.2  AR: 55 (Right) = 8.3±4.3  AR: 55 (Left) = 7.1±3.7  AR: 60 (Right) = 7.6±3.8  AR: 60 (Left) = 6.3±3.2  AR: 65 (Right) = 6.8±3.2  AR: 65 (Left) = 5.6±2.6 | AR: 20 (Right) = 0.68  AR: 20 (Left) = 0.85  AR: 25 (Right) = 0.72  AR: 25 (Left) = 0.86  AR: 30 (Right) = 0.75  AR: 30 (Left) = 0.86  AR: 35 (Right) = 0.79  AR: 35 (Left) = 0.85  AR: 40 (Right) = 0.81  AR: 40 (Left) = 0.86  AR: 45 (Right) = 0.85  AR: 45 (Left) = 0.85  AR: 50 (Right) = 0.89  AR: 50 (Left) = 0.86  AR: 55 (Right) = 0.93  AR: 55 (Left) = 8.6  AR: 60 (Right) = 0.97  AR: 60 (Left) = 0.86  AR: 65 (Right) = 1.05  AR: 65 (Left) = 0.85 |
| VanHarlinger, et al., 2015 | Isometric | Kg | Males:  AR: 20-24 = 15.2±4.5  AR: 25-29 = 19.3±3.7  AR: 30-34 = 18.3±6.4  AR: 35-39 = 14.8±3.7  AR: 40-44 = 19±4.1  AR: 45-49 = 14.7±4.7  AR: 50-54 = 16.4±6.4  AR: 55-59 = 18.5±5  AR: 60-64 = 15.1±2.8  Females:  AR: 20-24 = 9±2.1  AR: 25-29 = 8.6±3.4  AR: 30-34 = 7.5±3  AR: 35-39 = 8.8±3.5  AR: 40-44 = 8.8±3.4  AR: 45-49 = 10.3±4.2  AR: 50-54 = 7.9±3.7  AR: 55-59 = 8±2.6  AR: 60-64 = 7.8±2.9 | AR: 20-24 = 1.38  AR: 25-29 = 2.89  AR: 30-34 = 1.69  AR: 35-39 = 1.62  AR: 40-44 = 2.49  AR: 45-49 = 0.94  AR: 50-54 = 1.33  AR: 55-59 = 2.10  AR: 60-64 = 2.61 |
| Collins and O'Sullivan, 2018 | Isometric | N | Males:  87.8±21  Females:  49.3±12.30 | 1.83 |
| Lorenzo and Nunez, 2021 | Isometric | Kg | Males:  1.61±0.83  Females:  0.89±0.39 | 0.87 |
| Huberman, et al., 2020 | Isometric | Ibs | Males:  43.63±16.14  Females:  47.09±16.19 | 0.21 |
| Holzbaur, et al., 2007 | Isometric | Nm | Males:  74.4±10.8  Females:  34.9±5.4 | 2.55 |
| Westrick, et al., 2013 | Isometric | N/kg | Males:  0.35±0.08  Females:  0.29±0.10 | 0.75 |
| Hughes, et al., 1999 | Isometric | Nm | Males (Abducted 30°):  AR: 20-29 = 46±15  AR: 30-39 = 39±10  AR: 40-49 = 41±8  AR: 50-59 = 40±12  AR: 60+ = 30±14  Males (Abducted 60°):  AR: 20-29 = 40±14  AR: 30-39 = 34±8  AR: 40-49 = 37±10  AR: 50-59 = 37±10  AR: 60+ = 23±12  Males (Abducted 90°):  AR: 20-29 = 32±11  AR: 30-39 = 30±12  AR: 40-49 = 31±7  AR: 50-59 = 28±8  AR: 60+ = 22±11  Females (Abducted 30°):  AR: 20-29 = 23±9  AR: 30-39 = 26±9  AR: 40-49 = 25±8  AR: 50-59 = 24±5  AR: 60+ = 15±8  Females (Abducted 60°):  AR: 20-29 = 17±6  AR: 30-39 = 22±9  AR: 40-49 = 18±6  AR: 50-59 = 19±7  AR: 60+ = 10±6  Females (Abducted 90°):  AR: 20-29 = 15±5  AR: 30-39 = 19±8  AR: 40-49 = 14±6  AR: 50-59 = 14±7  AR: 60+ = 8±6 | Abducted 30°:  AR: 20-29 = 1.53  AR: 30-39 = 1.3  AR: 40-49 = 2  AR: 50-59 = 1.33  AR: 60+ = 1.07  Abducted 60°:  AR: 20-29 = 2.14  AR: 30-39 = 1.5  AR: 40-49 = 1.9  AR: 50-59 = 1.8  AR: 60+ = 1.08  Abducted 90°:  AR: 20-29 = 1.55  AR: 30-39 = 0.92  AR: 40-49 = 1.9  AR: 50-59 = 1.75  AR: 60+ = 1.27 |
| Magnusson, et al., 1995 | Isometric | Nm/kg | Males:  Left = 0.56±0.05  Right = 0.56±0.05  Females:  Left = 0.71±0.05  Right = 0.69±0.06 | Left = 3  Right = 2.17 |
| Guirelli, et al., 2021 | Isometric | N/lg | Males:  2.52±0.51  Females:  1.73±0.39 | 1.55 |
| Douma, et al., 2014 | Isometric | Nm | Males:  AR: 20-29 = 172±48  AR: 30-39 = 181±38  AR: 40-49 = 173±43  AR: 50-59 = 178±39  Females:  AR: 20-29 = 115±19  AR: 30-39 = 116±26  AR: 40-49 = 119±28  AR: 50-59 = 114±22 | AR: 20-29 = 1.19  AR: 30-39 = 1.71  AR: 40-49 = 1.26  AR: 50-59 = 1.64 |
| Andrews, et al., 1996 | Isometric | N | Males:  AR 50-59 = 237.9±55.5  AR 60-69 = 200.5±45.7  Females:  AR 50-59 = 135.2±24.4  AR 60-69 = 125±25.8 | AR 50-59 = 1.85  AR 60-69 = 1.65 |
| Alizadehkhaiyat, et al., 2014 | Isometric | N | Males:  98.8±29.2  Females:  60.1±13.0 |  |
| Backman, et al., 1995 | Isometric | N | Males:  AR: 20-30 = 141±33  AR: 30-40 = 132±36  AR: 40-50 = 137±27  AR: 50-60 = 135±23  AR: 60-70 = 119±27  Females:  AR: 20-30 = 84±13  AR: 30-40 = 96±20  AR: 40-50 = 80±13  AR: 50-60 = 83±22  AR: 60-70 = 69±17 | AR: 20-30 = 1.73  AR: 30-40 = 1  AR: 40-50 = 2.11  AR: 50-60 = 2.26  AR: 60-70 = 1.85 |
| Marcondes, et al., 2019 | Isokinetic:  60°/s  180°/s | Percent Body Mass | Males:  60°/s = 86.1±2.5  180°/s = 163.5±25.4  Females:  60°/s = 71±11.9  180°/s = 110.1±18 | 60°/s = 6.04  180°/s = 2.10 |
| Cahalan, et al., 1989 | Isokinetic:  60°/s  180°/s  300°/s | N, Nm | Males:  N = 52±11.5  60°/s = 39±9  180°/s = 32±8  300°/s = 26±7.5  Females:  N = 27±6.5  60°/s = 19±4  180°/s = 12.5±4  300°/s = 7.5±3 | N = 0.17  60°/s = 1.33  180°/s = 2.44  300°/s = 2.47 |
| Shklar and Dvir, 1995 | Isokinetic:  60°/s  120°/s  180°/s | Nm | Males:  Con. 60° = 50.5±13  Con. 120° = 46.9±13.3  Con. 180° = 43.6±11.9  Ecc. 60° = 64.8±18.2  Ecc. 120° = 67.9±17.3  Ecc. 180° = 73.1±18.4  Females:  Con. 60° = 28.4±4.6  Con. 120° = 26.4±4.2  Con. 180° = 24.8±3.5  Ecc. 60° = 37.3±6.1  Ecc. 120° = 38.9±7.5  Ecc. 180° = 41.8±7.2 | Con. 60° = 1.7  Con. 120° = 1.54  Con. 180° = 37.8  Ecc. 60° = 1.51  Ecc.120° = 1.68  Ecc. 180° = 1.70 |
| Ivey, et al., 1985 | Isokinetic:  60°/s  180°/s | Foot-Pounds | Males:  Slow = 41.6±11.4  Fast = 31.2±10.3  Females:  Slow = 21.6±6.6  Fast = 15.5±5.1 | Slow = 1.75  Fast = 1.52 |
| Reid, et al., 1989 | Isokinetic:  60°/s | Nm | Males:  50±14  Females:  23±5 | 1.93 |
| McMaster, et al., 1992 | Isokinetic:  30°/s  180°/s | Foot-Pounds | Males:  Con. 30° (Left) = 52.7±10.9  Con. 30° (Right) = 54±11.6  Con. 180° (Left) = 55.9±11.7  Con. 180° (Right) = 47.6±11.4  Females:  Con. 30° (Left) = 40.3±7.1  Con. 30° (Right) = 39.9±6.4  Con. 180° (Left) = 38±5.8  Con. 180° (Right) = 39±6.6 | Con. 30° (Left) = 1.14 Con. 30° (Right) = 1.22  Con. 180° (Left) = 1.53 Con. 180° (Right) = 0.75 |
| Sanchez, et al., 1999 | Isokinetic:  60°/s  120°/s | Nm | Males:  60°/s (Right) = 56.35±9.6  120°/s (Right) = 50.54±9.4  60°/s (Left) = 48.21±11.0  120°/s (Left) = 44.12±7.5  Females:  60°/s (Right) = 25.00±4.2  120°/s (Right) = 23.98±3.8  60°/s (Left) = 28.66±4.0  120°/s (Left) = 26.28±4.2 | 60°/s (Right) = 3.27  120°/s (Right) = 2.83  60°/s (Left) = 1.78  120°/s (Left) = 2.38 |
| Sanchez, et al. 2000 | Isokinetic:  60°/s  120°/s | Nm | Males:  60°/s (Right) = 56.35±9.6  120°/s (Right) = 50.54±9.4  60°/s (Left) = 52.28±10.7  120°/s (Left) = 47.75±10.5  Females:  60°/s (Right) = 25.01±4.2  120°/s (Right) = 23.98±3.8  60°/s (Left) = 24.02±5.7  120°/s (Left) = 23.98±3.8 | 60°/s (Right) = 3.26  120°/s (Right) = 2.83  60°/s (Left) = 2.64  120°/s (Left) = 2.26 |
| VanMeeteren, et al., 2002 | Isokinetic:  60°/s  120°/s  180°/s | Nm | Males:  63.15±17.1  Females:  36.65±8.05 | 0.77 |
| Murgia, et al., 2018 | Isokinetic:  60°/s  90°/s | Nm | Males:  Young 60°/s = 0.72±0.21  Young 90°/s = 0.72±0.21  Old 60°/s = 0.31±0.16  Old 90°/s = 0.29±0.16  Females:  Young 60°/s = 0.60±0.15  Young 90°/s = 0.61±0.11  Old 60°/s = 0.37±0.21  Old 90°/s = 0.34±0.18 | Young 60°/s = 0.57  Young 90°/s = 0.52  Old 60°/s = 0.29  Old 90°/s = 0.28 |
| Mayer, et al., 1994 | Isometric; Isokinetic:  Con. 300°/s  Con. 240°/s  Con. 180°/s  Con. 60°/s  Ecc. 60°/s  Ecc. 120°/s  Ecc. 180°/s  Ecc. 240°/s | Nm | Males:  ISO. = 47±12  IKO. Con. 300° = 30±8  IKO. Con. 240° = 31±7  IKO. Con. 180° = 33±8  IKO. Con. 60° = 38±7  IKO. Ecc. 60° = 41±10  IKO. Ecc. 120° = 45±10  IKO. Ecc. 180° = 44±10  IKO. Ecc. 240° = 44±8  Females:  ISO. = 28±6  IKO. Con. 300° = 18±4  IKO. Con. 240° = 19±4  IKO. Con. 180° = 18±4  IKO. Con. 60° = 22±4  IKO. Ecc. 60° = 25±4  IKO. Ecc. 120° = 29±6  IKO. Ecc. 180° = 31±5  IKO. Ecc. 240° = 29±6 | ISO.: 1.58  IKO. Con. 300° = 1.5  IKO. Con. 240° = 1.4  IKO. Con. 180° = 1.88  IKO. Con. 60° = 2.29  IKO. Ecc. 60° = 1.6  IKO. Ecc. 120° = 1.6  IKO. Ecc. 180° = 1.3  IKO. Ecc. 240° = 1.88 |
| Danneskiold-Samsoe, et al., 2009 | Isometric; Isokinetic:  30°/s  60°/s  90°/s  120°/s | N, Nm | Males (Nm):  AR: 20-29 = 46.0±9.3 (60 °/s), 45.7±9.9 (90 °/s), 44.1±10.1 (120 °/s)  AR: 30-39 = 46.8±7.0 (60 °/s), 45.6±5.2 (90 °/s), 44.2±5.0 (120 °/s)  AR: 40-49 = 42.4±8.1 (60 °/s), 41.0±8.2 (90 °/s), 39.2±6.8 (120 °/s)  AR: 50-59 = 47.3±8.6 (60 °/s), 45.7±8.1 (90 °/s), 41.1±7.6 (120 °/s)  AR: 60-69 = 38.4±10.2 (60 °/s), 36.2±10.1 (90 °/s), 34.3±7.3 (120 °/s)  AR: 70-79 = 38.8±6.1 (60 °/s), 34.6±7.1 (90 °/s), 33.4±7.2 (120 °/s)  Males (N):  AR: 20-29 = 60.2±14.0  AR: 30-39 = 59.1±8.0  AR: 40-49 = 54.5±14.2  AR: 50-59 = 59.1±9.6  AR: 60-69 = 47.9±11.4  AR: 70-79 = 49.3±11.3  Females (Nm):  AR: 20-29 = 28.3±5.8 (60 °/s), 25.6±5.9 (90 °/s), 24.8±4.8 (120 °/s)  AR: 30-39 = 28.9±7.8 (60 °/s), 27.2±7.5 (90 °/s), 26.2±7.0 (120 °/s)  AR: 40-49 = 33.2±8.7 (60 °/s), 31.0±8.8 (90 °/s), 28.7±6.7 (120 °/s)  AR: 50-59 = 28.3±5.3 (60 °/s), 26.2±5.2 (90 °/s), 26.1±4.2 (120 °/s)  AR: 60-69 = 22.5±5.2 (60 °/s), 22.7±5.3 (90 °/s), 22.5±4.3 (120 °/s)  AR: 70-79 = 21.4±4.4 (60 °/s), 21.0±3.7 (90 °/s), 21.1±4.3 (120 °/s)  Females (N):  AR: 20-29 = 30.9±7.4  AR: 30-39 = 32.9±8.8  AR: 40-49 = 36.2±7.4  AR: 50-59 = 32.9±6.8  AR: 60-69 = 27.1±6.3  AR: 70-79 = 25.9±6.2 | Nm:  AR: 20-29 = 1.90 (60 °/s), 2.03 (90 °/s), 1.91 (120 °/s)  AR: 30-39 = 2.56 (60 °/s), 3.54 (90 °/s), 3.6 (120 °/s)  AR: 40-49 = 1.14 (60 °/s), 1.22 (90 °/s), 1.54 (120 °/s)  AR: 50-59 = 2.21 (60 °/s), 2.41 (90 °/s), 1.97 (120 °/s)  AR: 60-69 = 1.56 (60 °/s), 1.34 (90 °/s), 1.62 (120 °/s)  AR: 70-79 = 2.85 (60 °/s), 1.92 (90 °/s), 1.71 (120 °/s)  N:  AR: 20-29 = 2.09  AR: 30-39 = 3.28  AR: 40-49 = 1.29  AR: 50-59 = 2.73  AR: 60-69 = 1.82  AR: 70-79 = 2.07 |
| Harbo, et al., 2012 | Isometric;  Isokinetic:  60°/s | Nm | Males:  AR: <30 = IKO: 57±12; ISO: 60±14  AR: 30-39 = IKO: 67±10; ISO: 70±9  AR: 40-49 = IKO: 63±10; ISO: 67±11  AR: 50-59 = IKO: 62±12; ISO: 64±14  AR: 60-69 = IKO: 57±11; ISO: 58±16  Females:  AR: <30 =IKO: 42±8; ISO: 43±13  AR: 30-39 = IKO: 40±9; ISO: 41±9  AR: 40-49 = IKO: 37±8; ISO: 38±7  AR: 50-59 = IKO: 39±7; ISO: 39±7  AR: 60-69 = IKO: 32±5; ISO: 31±8 | AR: <30 = IKO: 1.25; ISO: 1.21  AR: 30-39 = IKO: 2.7; ISO: 3.22  AR: 40-49 = IKO: 2.6; ISO: 2.64  AR: 50-59 = IKO: 1.92; ISO: 1.79  AR: 60-69 = IKO: 2.27; ISO: 1.69 |
