# Supplementary material for: Sex differences in strength at the shoulder: a systematic review
Source: PeerJ. 2024 Mar 20;12:e16968. doi: 10.7717/peerj.16968 (PMC10960529; doi:10.7717/peerj.16968)
Supplement: Supplemental Information 4 — Isometric (ISO) and isokinetic (IKO) data of concentric (Con) and Eccentric (Ecc) movement types. Age ranges (AR) included. Outcomes are relative to the described measurement unit; where available, effect sizes were extracted or calculated (Cohen’s d). [file peerj-12-16968-s004.docx]

# **Supplementary Table 3: Extracted data for studies with shoulder adduction data.**

Isometric (ISO) and isokinetic (IKO) data of concentric (Con) and Eccentric (Ecc) movement types. Age ranges (AR) included. Outcomes are relative to the described measurement unit; where available, effect sizes were extracted or calculated (Cohen's d).

| **Title** | **Movement Type** | **Measurement Unit** | **Outcomes** | **Effect Size (Cohen’s d)** |
| --- | --- | --- | --- | --- |
| Murray, et al., 1985 | Isometric | kg-cm | Males:  Young 45° = 1051±59  Old 45° = 833±49  Females:  Young 45° = 561±33  Old 45° = 387±25 | Young 45° = 8.31  Old 45° = 9.10 |
| Meldrum, et al., 2007 | Isometric | Kg | Males:  AR: 20 (Right) = 33±21.6  AR: 20 (Left) = 31.8±21.4  AR: 25 (Right) = 32.4±21.3  AR: 25 (Left)= 31.2±21.1  AR: 30 (Right) = 31.9±21.1  AR: 30 (Left)= 30.7±20.8  AR: 35 (Right) = 31.3±20.8  AR: 35 (Left)= 30.1±20.5  AR: 40 (Right) = 30.7±20.5  AR: 40 (Left)= 29.5±20.3  AR: 45 (Right) = 30.2±20.3  AR: 45 (Left)= 29±20  AR: 50 (Right) = 29.6±20  AR: 50 (Left)= 28.4±19.7  AR: 55 (Right) = 29±19.7  AR: 55 (Left) = 27.9±19.5  AR: 60 (Right) = 28.5±19.4  AR: 60 (Left) = 27.3±19.2  AR: 65 (Right) = 27.9±19.2  AR: 65 (Left)= 26.7±18.9  Females:  AR: 20 (Right)= 19.5±12.1  AR: 20 (Left) = 18.3±11.8  AR: 25 (Right) = 18.9±11.8  AR: 25 (Left) = 17.7±11.5  AR: 30 (Right)= 18.3±11.5  AR: 30 (Left) = 17.1±11.3  AR: 35 (Right)= 17.8±11.2  AR: 35 (Left)= 16.6±11  AR: 40 (Right)= 17.2±11  AR: 40 (Left)= 16±10.7  AR: 45 (Right)= 16.6±10.7  AR: 45 (Left) = 15.5±10.2  AR: 50 (Right)= 16.1±10.4  AR: 50 (Left) = 14.9±10.2  AR: 55 (Right) = 15.5±10.2  AR: 55 (Left)= 14.3±9.9  AR: 60 (Right) = 15±9.9  AR: 60 (Left)= 13.8±9.6  AR: 65 (Right)= 14.4±9.6  AR: 65 (Left) = 13.2±9.6 | AR: 20 (Right) = 0.64  AR: 20 (Left) = 0.63  AR: 25 (Right)= 0.63  AR: 25 (Left) = 0.64  AR: 30 (Right)= 0.64  AR: 30 (Left) = 0.65  AR: 35 (Right)= 0.65  AR: 35 (Left) = 0.66  AR: 40 (Right) = 0.66  AR: 40 (Left)= 0.67  AR: 45 (Right) = 0.67  AR: 45 (Left)= 0.68  AR: 50 (Right) = 0.68  AR: 50 (Left)= 0.69  AR: 55 (Right) = 0.69  AR: 55 (Left) = 0.70  AR: 60 (Right) = 0.70  AR: 60 (Left) = 0.70  AR: 65 (Right) = 0.70  AR: 65 (Left) = 0.71 |
| Huberman, et al., 2020 | Isometric | lbs | Males:  44.93±16.09  Females:  48.20±16.15 | 0.20 |
| Holzbaur, et al., 2007 | Isometric | Nm | Males:  93.7±11.3  Females:  42.1±5.4 | 4.57 |
| Hughes, et al., 1999 | Isometric | Nm | Males (Abducted 30°):  AR: 20-29 = 74±21  AR: 30-39 = 67±10  AR: 40-49 = 67±12  AR: 50-59 = 61±12  AR: 60+ = 54±13  Males (Abducted 60°):  AR: 20-29 = 84±22  AR: 30-39 = 80±15  AR: 40-49 = 75±11  AR: 50-59 = 75±13  AR: 60+ = 65±12  Males (Abducted 90°):  AR: 20-29 = 79±21  AR: 30-39 = 79±18  AR: 40-49 = 71±11  AR: 50-59 = 72±14  AR: 60+ = 62±14  Females (Abducted 30°):  AR: 20-29 = 37±16  AR: 30-39 = 41±14  AR: 40-49 = 37±12  AR: 50-59 = 35±9  AR: 60+ = 27±8  Females (Abducted 60°):  AR: 20-29 = 43±15  AR: 30-39 = 49±14  AR: 40-49 = 43±11  AR: 50-59 = 41±11  AR: 60+ = 30±11  Females (Abducted 90°):  AR: 20-29 = 39±12  AR: 30-39 = 49±14  AR: 40-49 = 38±10  AR: 50-59 = 40±10  AR: 60+ = 31±11 | Abducted 30°:  AR: 20-29 = 1.76  AR: 30-39 = 2.6  AR: 40-49 = 2.5  AR: 50-59 = 0.33  AR: 60+ = 2.08  Abducted 60°:  AR: 20-29 = 1.86  AR: 30-39 = 2.07  AR: 40-49 = 2.91  AR: 50-59 = 2.62  AR: 60+ = 2.92  Abducted 90°:  AR: 20-29 = 1.90  AR: 30-39 = 1.67  AR: 40-49 = 3  AR: 50-59 = 2.29  AR: 60+ = 0.14 |
| Marcondes, et al., 2019 | Isokinetic:  60°/s  180°/s | Percent Body Mass | Males:  60°/s = 111.3±10.9  180°/s = 183.3±36.4  Females:  60°/s = 76.2±5.8  180°/s = 114.5±12.4 | 60°/s = 3.22  180°/s = 1.89 |
| Cahalan, et al., 1989 | Isokinetic:  60°/s  180°/s  300°/s | N, Nm | Males:  N = 72±15  60°/s = 79.5±16  180°/s = 72.5±14  300°/s = 64.5±13  Females:  N = 39.5±9  60°/s = 37.5±6.5  180°/s = 32.5±7.5  300°/s = 28±8 | N = 2.17  60°/s = 2.63  180°/s = 2.86  300°/s = 2.81 |
| Shklar and Dvir, 1995 | Isokinetic:  60°/s  120°/s  180°/s | Nm | Males:  Con. 60° = 72.9±19.5  Con. 120° = 69.8±15.2  Con. 180° = 66.1±17.4  Ecc. 60° = 95.2±28  Ecc. 120° = 92.7±27.8  Ecc. 180° = 97.5±32.9  Females:  Con. 60° = 32.4±6.9  Con. 120° = 31±5.3  Con. 180° = 29.4±4.4  Ecc. 60° = 46.7±8.9  Ecc. 120° = 47.5±9  Ecc. 180° = 50.1±8.2 | Con. 60° = 2.08  Con. 120° = 2.55  Con. 180° = 2.11  Ecc. 60° = 1.73  Ecc. 120° = 1.63  Ecc. 180° = 1.19 |
| Ivey, et al., 1985 | Isokinetic:  60°/s  180°/s | Foot-Pounds | Males:  Slow = 65.9±16.4  Fast = 55.5±17.0  Females:  Slow = 37.2±6.7  Fast = 30.7±31.6 | Slow = 1.75  Fast = 1.46 |
| Reid, et al., 1989 | Isokinetic:  60°/s | Nm | Males:  86±19  Females:  46±9 | 2.11 |
| McMaster, et al., 1992 | Isokinetic:  30°/s  180°/s | Foot-Pounds | Males:  Con. 30° (Left) = 38.2±7.8  Con. 30° (Right) = 35.3±7.7  Con. 180° (Left) = 35.2±7.2  Con. 180° (Right) = 32.6±7.8  Females:  Con. 30° (Left) = 25.4±3.9  Con. 30° (Right) = 24.2±3.8  Con. 180° (Left) = 22±5.9  Con. 180° (Right) = 22.5±4 | Con. 30° (Left) = 1.64  Con. 30° (Right) = 1.44  Con. 180° (Left) = 1.83  Con. 1800° (Right) = 1.29 |
| Sanchez, et al., 1999 | Isokinetic:  60°/s  120°/s | Nm | Males:  60°/s (Right) = 77.63±12.5  120°/s (Right) = 75.43±9.2  60°/s (Left) = 66.31±16.2  120°/s (Left) = 66.21±15.6  Females:  60°/s (Right) = 34.52±8.3  120°/s (Right) = 33.91±7.9  60°/s (Left) = 39.25±7.9  120°/s (Left) = 37.90±8.0 | 60°/s (Right) = 3.45  120°/s (Right) = 4.51  60°/s (Left) = 1.67  120°/s (Left) = 1.81 |
| Sanchez, et al. 2000 | Isokinetic:  60°/s  120°/s | Nm | Males:  60°/s (Right) = 77.63±12.5  120°/s (Right) = 75.43±9.2  60°/s (Left) = 64.29±9.6  120°/s (Left) = 65.65±8.4  Females:  60°/s (Right) = 34.52±8.3  120°/s (Right) = 33.91±7.9  60°/s (Left) = 31.26±6.0  120°/s (Left) = 33.91±7.9 | 60°/s (Right) = 3.45  120°/s (Right) = 4.51  60°/s (Left) = 3.44  120°/s (Left) = 3.78 |
| VanMeeteren, et al., 2002 | Isokinetic:  60°/s  120°/s  180°/s | Nm | Males:  68.6±14.95  Females:  39.35±8.85 | 1.96 |
| Harbo, et al., 2012 | Isokinetic:  60°/s | Nm | Males:  AR: <30 = 77±14  AR: 30-39 = 92±16  AR: 40-49 = 88±23  AR: 50-59 = 84±14  AR: 60-69 = 83±18  Females:  AR: <30 = 46±14  AR: 30-39 = 50±9  AR: 40-49 = 46±10  AR: 50-59 = 45±9  AR: 60-69 = 41±7 | AR: <30 = 2.21  AR: 30-39 = 2.63  AR: 40-49 = 1.83  AR: 50-59 = 2.79  AR: 60-69 = 2.33 |
| Mayer, et al., 1994 | Isometric; Isokinetic:  Con. 300°/s  Con. 240°/s  Con. 180°/s  Con. 60°/s  Ecc. 60 °/s  Ecc. 120 °/s  Ecc. 180 °/s  Ecc. 240 °/s | Nm | Males:  ISO. = 72±17  IKO. Con. 300° = 45±18  IKO. Con. 240° = 48±17  IKO. Con. 180° = 48±13  IKO. Con. 60° = 53±11  IKO. Ecc. 60° = 55±11  IKO. Ecc. 120° = 59±10  IKO. Ecc. 180° = 61±14  IKO. Ecc. 240° = 63±14  Females:  ISO. = 34±9  IKO. Con. 300° = 23±7  IKO. Con. 240° = 24±7  IKO. Con. 180° = 22±6  IKO. Con. 60° = 29±8  IKO. Ecc. 60° = 31±8  IKO. Ecc. 120° = 36±10  IKO. Ecc. 180° = 39±11  IKO. Ecc. 240° = 39±9 | ISO. = 2.24  IKO. Con. 300° = 1.88  IKO. Con. 240° = 2.12  IKO. Con. 180° = 2.12  IKO. Con. 60° = 2.18  IKO. Ecc. 60° = 2.18  IKO. Ecc. 120° = 2.3  IKO. Ecc. 180° = 1.57  IKO. Ecc. 240° = 1.71 |
| Danneskiold-Samsoe, et al., 2009 | Isometric; Isokinetic:  30 °/s  60 °/s  90 °/s  120 °/s | N, Nm | Males (Nm):  AR: 20-29 = 64.7±16.2 (60 °/s), 61.2±16.7 (90 °/s), 61.1±17.7 (120 °/s)  AR: 30-39 = 56.0±9.6 (60 °/s), 54.1±13.2 (90 °/s), 51.4±11.3 (120 °/s)  AR: 40-49 = 53.0±16.1 (60 °/s), 51.1±13.6 (90 °/s), 49.0±14.8 (120 °/s)  AR: 50-59 = 53.3±16.3 (60 °/s), 47.0±10.8 (90 °/s), 44.9±11.3 (120 °/s)  AR: 60-69 = 48.2±14.0 (60 °/s), 44.8±11.1 (90 °/s), 43.2±10.1 (120 °/s)  AR: 70-79 = 46.8±7.5 (60 °/s), 43.9±7.0 (90 °/s), 42.3±7.8 (120 °/s)  Males (N):  AR: 20-29 = 89.6±26.9  AR: 30-39 = 78.9±20.9  AR: 40-49 = 77.9±17.4  AR: 50-59 = 75.9±8.5  AR: 60-69 = 69.4±18.5  AR: 70-79 = 68.1±12.9  Females (Nm):  AR: 20-29 = 33.3±6.5 (60 °/s), 30.2±5.3 (90 °/s), 28.2±3.9 (120 °/s)  AR: 30-39 = 32.3±7.7 (60 °/s), 32.4±7.6 (90 °/s), 29.6±6.9 (120 °/s)  AR: 40-49 = 34.3±8.8 (60 °/s), 32.1±7.6 (90 °/s), 30.6±8.3 (120 °/s)  AR: 50-59 = 29.4±4.7 (60 °/s), 26.3±6.1 (90 °/s), 26.7±6.4 (120 °/s)  AR: 60-69 = 25.7±6.0 (60 °/s), 24.8±5.9 (90 °/s), 25.3±6.1 (120 °/s)  AR: 70-79 = 24.3±4.6 (60 °/s), 21.8±3.8 (90 °/s), 21.7±4.2 (120 °/s)  Females (N):  AR: 20-29 = 42.0±7.4  AR: 30-39 = 47.0±9.5  AR: 40-49 = 47.4±12.5  AR: 50-59 = 41.9±7.4  AR: 60-69 = 39.8±9.9  AR: 70-79 = 34.0±6.6 | Nm:  AR: 20-29 = 1.94 (60 °/s), 1.86 (90 °/s), 1.86 (120 °/s)  AR: 30-39 = 2.47 (60 °/s), 1.64 (90 °/s), 1.93 (120 °/s)  AR: 40-49 = 1.16 (60 °/s), 1.39 (90 °/s), 1.24 (120 °/s)  AR: 50-59 = 1.47 (60 °/s), 1.92 (90 °/s), 1.61 (120 °/s)  AR: 60-69 = 1.61 (60 °/s), 1.80 (90 °/s), 1.77 (120 °/s)  AR: 70-79 = 3 (60 °/s), 3.16 (90 °/s), 2.64 (120 °/s)  N:  AR: 20-29 = 1.77  AR: 30-39 = 1.53  AR: 40-49 = 1.75  AR: 50-59 = 4  AR: 60-69 = 1.6  AR: 70-79 = 2.64 |
