# Supplementary material for: Sex differences in strength at the shoulder: a systematic review
Source: PeerJ. 2024 Mar 20;12:e16968. doi: 10.7717/peerj.16968 (PMC10960529; doi:10.7717/peerj.16968)
Supplement: Supplemental Information 5 — Isometric (ISO) and isokinetic (IKO) data of concentric (Con) and Eccentric (Ecc) movement types. Age ranges (AR) included. Outcomes are relative to the described measurement unit; where available, effect sizes were extracted or calculated (Cohen’s d). [file peerj-12-16968-s005.docx]

# **Supplementary Table 4: Extracted data for studies with shoulder scaption data.**

Isometric (ISO) and isokinetic (IKO) data of concentric (Con) and Eccentric (Ecc) movement types. Age ranges (AR) included. Outcomes are relative to the described measurement unit; where available, effect sizes were extracted or calculated (Cohen's d).

| **Title** | **Movement Type** | **Measurement Unit** | **Outcomes** | **Effect Size (Cohen’s d)** |
| --- | --- | --- | --- | --- |
| Alizadehkhaiyat, et al., 2014 | Isometric | N | Males:  99.7±27.0  Females:  60.4±11.8 | 1.46 |
| Eren, et al., 2019 | Isometric | N | Males:  Empty Can = 88.92±23.96  Palm Down = 98.89±25.99  Females:  Empty Can = 54.95±14.18  Palm Down = 54.26±10.8 | Empty Can = 1.42  Palm Down = 1.72 |
