# Supplementary material for: Sex differences in strength at the shoulder: a systematic review
Source: PeerJ. 2024 Mar 20;12:e16968. doi: 10.7717/peerj.16968 (PMC10960529; doi:10.7717/peerj.16968)
Supplement: Supplemental Information 6 — Isometric (ISO) and isokinetic (IKO) data of concentric (Con) and Eccentric (Ecc) movement types. Age ranges (AR) included. Outcomes are relative to the described measurement unit; where available, effect sizes were extracted or calculated (Cohen’s d). [file peerj-12-16968-s006.docx]

# **Supplementary Table 5: Extracted data for studies with shoulder flexion data.**

Isometric (ISO) and isokinetic (IKO) data of concentric (Con) and Eccentric (Ecc) movement types. Age ranges (AR) included. Outcomes are relative to the described measurement unit; where available, effect sizes were extracted or calculated (Cohen's d).

| **Title** | **Movement Type** | **Measurement Unit** | **Outcomes** | **Effect Size (Cohen’s d)** |
| --- | --- | --- | --- | --- |
| Murray, et al., 1985 | Isometric | kg-cm | Males:  Young 0° = 1058±47  Young 45° = 566±24  Old 0° = 852±42  Old 45° = 478±23  Females:  Young 0° = 514±17  Young 45° = 338±16  Old 0° = 384±29  Old 45° = 224±18 | Young 0° = 11.57  Young 45° = 9.5  Old 0° = 11.14  Old 45° = 11.04 |
| Yates, et al., 1980 | Isometric | N | Males:  45° = 125.5±22  135° = 95.1±21  Females:  45° = 59.8±19  135° = 44.1±14 | 45° = 2.98  135° = 2.43 |
| Lannersten, et al., 1993 | Isometric | Nm | Males:  AR: 19-34 = 52.7±13  AR: 35-44 = 51.7±12  AR: 45-65 = 46.8±10.7  Females:  AR: 19-34 = 26.5±5.6  AR: 35-44 = 29.4±6.2  AR: 45-65 = 26.8±6.1 | AR: 19-34 = 2.02  AR: 35-44 = 1.86  AR: 45-65 = 1.87 |
| Barnekow-Bergkvist, et al., 2007 | Isometric | N, Nm | Males:  1420.0±220.0  Females:  940.0±210.0 | 2.18 |
| MacDonell and Keir, 2005 | Isometric | Nm | Males:  30° = 86.9±7.1  60° = 88.5±5.6  90° = 79.3±6.9  Females:  30° = 43.1±3.5  60° = 42.8±2.8  90° = 42.3±2.7 | 30° = 6.17  60° = 8.16  90° = 5.36 |
| VanHarlinger, et al., 2015 | Isometric | Kg | Males:  AR: 20-24 = 19.6±4.6  AR: 25-29 = 22.4±4.7  AR: 30-34 = 22.8±5.9  AR: 35-39 = 20±4.9  AR: 40-44 = 23.9±3.9  AR: 45-49 = 18.3±4.4  AR: 50-54 = 18.6±6.28  AR: 55-59 = 20.3±4.6  AR: 60-64 = 18.4±3.7  Females:  AR: 20-24 = 10.3±3.2  AR: 25-29 = 9.8±3.5  AR: 30-34 = 9.5±2.6  AR: 35-39 = 10.4±4.7  AR: 40-44 = 11.2±3.6  AR: 45-49 = 12.1±3.9  AR: 50-54 = 11±4  AR: 55-59 = 9.4±3.5  AR: 60-64 = 9.2±2.5 | AR: 20-24 = 2.02  AR: 25-29 = 2.69  AR: 30-34 = 2.25  AR: 35-39 = 1.96  AR: 40-44 = 3.26  AR: 45-49 = 1.41  AR: 50-54 = 1.21  AR: 55-59 = 2.37  AR: 60-64 = 2.49 |
| Ferreira, et al., 2020 | Isometric | Nm/kg | Male:  Right = 0.83±0.12  Left = 0.82±0.13  Female:  Right = 0.72±0.09  Left = 0.62±0.08 | Right = 0.92  Left = 1.54 |
| Huberman, et al., 2020 | Isometric | Ibs | Male:  36.93±10.82  Female:  39.39±10.85 | 0.23 |
| Westrick, et al., 2013 | Isometric | N/kg | Males:  0.12±0.03  Females:  0.10±0.03 | 0.67 |
| Hughes, et al., 1999 | Isometric | Nm | Males:  Flexed 30°:  AR: 20-29 = 63±14  AR: 30-39 = 55±15  AR: 40-49 = 52±11  AR: 50-59 = 45±9  AR: 60+ = 45±12  Flexed 60°:  AR: 20-29 = 50±11  AR: 30-39 = 44±11  AR: 40-49 = 43±8  AR: 50-59 = 35±9  AR: 60+ = 36±11  Flexed 90°:  AR: 20-29 = 47±16  AR: 30-39 = 41±14  AR: 40-49 = 38±8  AR: 50-59 = 24±13  AR: 60+ = 28±13  Females:  Flexed 30°:  AR: 20-29 = 36±15  AR: 30-39 = 37±13  AR: 40-49 = 26±11  AR: 50-59 = 23±6  AR: 60+ = 16±7  Flexed 60°:  AR: 20-29 = 33±14  AR: 30-39 = 33±13  AR: 40-49 = 20±10  AR: 50-59 = 20±5  AR: 60+ = 14±5  Flexed 90°:  AR: 20-29 = 26±13  AR: 30-39 = 26±9  AR: 40-49 = 16±9  AR: 50-59 = 16±6  AR: 60+ = 12±6 | Flexion:  Flexed 30°:  AR: 20-29 = 1.93  AR: 30-39 = 1.20  AR: 40-49 = 2.36  AR: 50-59 = 2.44  AR: 60+ = 2.42  Flexed 60°:  AR: 20-29 = 1.55  AR: 30-39 = 1  AR: 40-49 = 2.88  AR: 50-59 = 1.67  AR: 60+ = 2)  Flexed 90°:  AR: 20-29 = 1.31  AR: 30-39 = 1.07  AR: 40-49 = 2.75  AR: 50-59 = 0.62  AR: 60+ = 1.23 |
| Pontillo and Sennett, 2020 | Isometric | kg | Males:  10.7±3.5  Females:  8±2.5 | 0.77 |
| Andrews, et al., 1996 | Isometric | N | Males:  AR: 50-59 = 267.7±46  AR: 60-69 = 231.8±42.4  Females:  AR: 50-59 = 161.6±30.4  AR: 60-69 = 147.5±30.9 | AR: 50-59 = 2.31  AR: 60-69 = 1.99 |
| Busko & Gajewski, 2011 | Isometric | Nm | Males:  279.55±19.90  Females:  163.95±44.8 | 5.81 |
| Alizadehkhaiyat, et al., 2014 | Isometric | N | Males:  105.2±25.6  Females:  63.0±12.4 | 1.65 |
| Marcondes, et al., 2019 | Isokinetic:  60°/s  180°/s | Percent Body Mass | Males:  60°/s: 102.3±7.4  180°/s: 172.1±17.9  Females:  60°/s: 72.4±8.2  180°/s: 112.9±20.2 | 60°/s = 4.04  180°/s = 3.31 |
| Cahalan, et al., 1991 | Isokinetic:  60°/s  180°/s  300°/s | N, Nm | Males:  N = 63±17.5  60°/s = 49±11.5  180°/s = 42±11  300°/s = 34.5±11  Females:  N = 27±8.5  60°/s 22±5  180°/s = 16±5.5  300°/s = 11.5±4.5 | N = 2.06  60°/s = 2.35  180°/s = 2.36  300°/s = 2.09 |
| Shklar and Dvir, 1995 | Isokinetic:  60°/s  120°/s  180°/s | Nm | Males:  Con. 60°/s = 61.2±13.3  Con. 120°/s = 57.1±9.8  Con. 180°/s = 52.8±10.3  Ecc. 60°/s = 72.4±18  Ecc. 120°/s = 75.2±18.4  Ecc.180°/s = 77.1±18.1  Females:  Con. 60°/s = 36.5±6.1  Con. 120°/s = 35.5±6.3  Con. 180°/s = 32.3±5.7  Ecc. 60°/s = 43.1±7.1  Ecc. 120°/s = 45.7±8.9  Ecc. 180°/s = 47.7±8.7 | Con. 60°/s = 1.86  Con. 120°/s = 2.20  Con. 180°/s = 1.99  Ecc. 60°/s = 1.63  Ecc. 120°/s = 1.60  Ecc. 180°/s = 1.62 |
| Koski and McGill, 1994 | Isokinetic:  50°/s | Nm | Males:  Peak Con. = 56.0±13.1  Peak Ecc. = 83.9±18.1  Ecc. 0° = 59.2±20.0  Con. 45° = 46.9±11.7  Ecc. 45° = 75.3±16.3  Females:  Peak Con. = 29.0±5.86  Peak Ecc. = 43.4±10.4  Ecc. 0° = 27.0±9.06  Con. 45° = 25.8±5.33  Ecc. 45° = 38.6±10.7 | Peak Con. = 2.06  Peak Ecc. = 2.24  Ecc. 0° = 1.61  Con. 45° = 1.80  Ecc. 45° = 2.25 |
| Khalaf and Parnianpour, 2001 | Isokinetic:  10°/s  50°/s  100°/s  150°/s  200°/s  250°/s | Nm | Males:  10°/s = 69.91±10.21  50°/s = 58.62±11.45  100°/s = 43.79±8.58  150°/s = 39.17±7.93  200°/s = 36.84±7.51  250°/s = 34.44±8.09  Females:  10°/s = 30.91±7.74  50°/s = 24.87±8.35  100°/s = 18.44±5.29  150°/s = 16.01±5  200°/s = 13.71±4.24  250°/s = 12.33±3.08 | 10°/s = 3.82  50°/s = 2.95  100°/s = 2.95  150°/s = 2.92  200°/s = 3.08  250°/s = 2.73 |
| Murgia, et al., 2018 | Isokinetic:  60°/s  90°/s | Nm | Males:  Young 60°/s = 0.86±0.22  Young 90°/s = 0.81±0.13  Old 60°/s = 0.42±0.21  Old 90°/s = 0.42±0.11  Females:  Young 60°/s = 0.50±0.13  Young 90°/s = 0.58±0.14  Old 60°/s = 0.36±0.07  Old 90°/s = 0.28±0.17 | Young 60°/s = 1.64  Young 90°/s = 1.77  Old 60°/s = 0.29  Old 90°/s = 1.27 |
| Mayer, et al., 1994 | Isometric;  Isokinetic:  Con. 300°/s  Con. 240°/s  Con. 180°/s  Con. 60°/s  Ecc. 60°/s  Ecc. 120°/s  Ecc. 180°/s  Ecc. 240°/s | Nm | Males:  ISO. = 68±18  IKO. Con. 300°/s = 39±9  IKO. Con. 240°/s = 42±9  IKO. Con. 180°/s = 41±8  IKO. Con. 60°/s = 41±8  IKO. Ecc. 60°/s = 56±13  IKO. Ecc. 120°/s = 60±14  IKO. Ecc. 180°/s = 56±11  IKO. Ecc. 240°/s = 52±8  Females:  ISO. = 45±13  IKO. Con. 300°/s = 24±5  IKO. Con. 240°/s = 24±5  IKO. Con. 180°/s = 25±5  IKO. Con. 60°/s = 29±5  IKO. Ecc. 60°/s = 32±6  IKO. Ecc. 120°/s = 40±13  IKO. Ecc. 180°/s = 40±2  IKO. Ecc. 240°/s = 34±2 | ISO. = 1.28  IKO. Con. 300°/s = 1.67  IKO. Con. 240°/s = 2  IKO. Con. 180°/s = 2  IKO. Con. 60°/s = 1.5  IKO. Ecc. 60°/s = 1.85  IKO. Ecc. 120°/s = 1.43  IKO. Ecc. 180°/s = 1.45  IKO. Ecc. 240°/s = 2.25 |
| Danneskiold-Samsoe, et al., 2009 | Isometric;  Isokinetic:  30°/s  60°/s  90°/s  120°/s | N, Nm | Males (Nm):  AR: 20-29 = 51.8±10.6 (60°/s), 50.9±12.4 (90°/s), 49.6±14.8 (120°/s)  AR: 30-39 = 54.7±7.3 (60°/s), 54.6±8.1 (90°/s), 52.9±7.2 (120°/s)  AR: 40-49 = 49.5±9.3 (60°/s), 48.0±8.0 (90°/s), 46.7±9.7 (120°/s)  AR: 50-59 = 48.4±9.1 (60°/s), 47.3±8.8 (90°/s), 43.9±6.4 (120°/s)  AR: 60-69 = 41.9±7.6 (60°/s), 41.6±8.4 (90°/s), 38.6±9.6 (120°/s)  AR: 70-79 = 44.1±8.5 (60°/s), 41.9±6.3 (90°/s), 41.7±6.8 (120°/s)  Males (N):  AR: 20-29 = 63.0±15.8  AR: 30-39 = 58.5±7.4  AR: 40-49 = 58.0±13.6  AR: 50-59 = 58.7±10.6  AR: 60-69 = 53.9±10.5  AR: 70-79 = 53.2±8.6  Females **(**Nm):  AR: 20-29 = 30.1±6.5 (60°/s), 27.6±4.3 (90°/s), 26.6±5.2 (120°/s)  AR: 30-39 = 31.7±7.6 (60°/s), 29.3±7.1 (90°/s), 27.0±5.9 (120°/s)  AR: 40-49 = 33.8±7.1 (60°/s), 30.5±6.2 (90°/s), 29.5±6.0 (120°/s)  AR: 50-59 = 29.1±6.0 (60°/s), 26.8±5.6 (90°/s), 25.8±4.6 (120°/s)  AR: 60-69 = 26.9±5.3 (60°/s), 24.7±4.0 (90°/s), 24.1±4.3 (120°/s)  AR: 70-79 = 24.3±5.6 (60°/s), 22.7±4.6 (90°/s), 22.2±4.7 (120°/s)  Females (N):  AR: 20-29 = 30.0±6.5  AR: 30-39 = 34.3±9.7  AR: 40-49 = 35.0±7.3  AR: 50-59 = 32.0±6.9  AR: 60-69 = 26.6±5.4  AR: 70-79 = 26.7±7.1 | Nm:  AR: 20-29 = 2.05 (60°/s), 1.88 (90°/s), 1.55 (120°/s)  AR: 30-39 = 3.15 (60°/s), 3.12 (90°/s), 3.59 (120°/s)  AR: 40-49 = 1.69 (60°/s), 2.19 (90°/s), 1.77 (120°/s)  AR: 50-59 = 2.12 (60°/s), 2.33 (90°/s), 2.83 (120°/s)  AR: 60-69 = 1.97 (60°/s), 2.01 (90°/s), 1.51 (120°/s)  AR: 70-79 = 2.33 (60°/s), 3.05 (90°/s), 2.87 (120°/s)  N:  AR: 20-29 = 2.09  AR: 30-39 = 3.27  AR: 40-49 = 1.69  AR: 50-59 = 2.51  AR: 60-69 = 2.6  AR: 70-79 = 3.08 |
