# Supplementary material for: Sex differences in strength at the shoulder: a systematic review
Source: PeerJ. 2024 Mar 20;12:e16968. doi: 10.7717/peerj.16968 (PMC10960529; doi:10.7717/peerj.16968)
Supplement: Supplemental Information 7 — Isometric (ISO) and isokinetic (IKO) data of concentric (Con) and Eccentric (Ecc) movement types. Age ranges (AR) included. Outcomes are relative to the described measurement unit; where available, effect sizes were extracted or calculated (Cohen’s d). [file peerj-12-16968-s007.docx]

# **Supplementary Table 6: Extracted data for studies with shoulder extension data.**

Isometric (ISO) and isokinetic (IKO) data of concentric (Con) and Eccentric (Ecc) movement types. Age ranges (AR) included. Outcomes are relative to the described measurement unit; where available, effect sizes were extracted or calculated (Cohen's d).

| **Title** | **Movement Type** | **Measurement Unit** | **Outcomes** | **Effect Size (Cohen’s d)** |
| --- | --- | --- | --- | --- |
| Murray, et al., 1985 | Isometric | kg-cm | Males:  Young 0° = 812±40  Old 0° = 755±30  Females:  Young 0° = 536±30  Old 0° = 359±23 | Young 0° = 6.9  Old 0° = 13.2 |
| VanHarlinger, et al., 2015 | Isometric | Kg | Males:  AR: 20-24 = 19.6±5.0  AR: 25-29 = 17.9±3.3  AR: 30-34 = 17.9±4.1  AR: 35-39 = 19.1±3.6  AR: 40-44 = 18.7±4.2  AR: 45-49 = 14.1±5.4  AR: 50-54 = 14.7±5.3  AR: 55-59 = 15.6±3.1  AR: 60-64 = 15.6±4.3  Females:  AR: 20-24 = 9.9±3.2  AR: 25-29 = 7±2.8  AR: 30-34 = 8.5±2.7  AR: 35-39 = 8.4±3.7  AR: 40-44 = 7.5±3.5  AR: 45-49 = 10.6±2.8  AR: 50-54 = 7.2±2.6  AR: 55-59 = 6.9±3.2  AR: 60-64 = 7.2±1.7 | AR: 20-24 = 1.94  AR: 25-29 = 3.30  AR: 30-34 = 2.85  AR: 35-39 = 2.97  AR: 40-44 = 2.67  AR: 45-49 = 0.65  AR: 50-54 = 1.42  AR: 55-59 = 2.81  AR: 60-64 = 2.49 |
| Ferreira, et al., 2020 | Isometric | Nm/kg | Males:  Right = 1.06±0.14  Left = 1.00±0.13  Females:  Right = 0.84±0.13  Left = 0.78±0.10 | Right = 1.57  Left = 1.69 |
| Huberman, et al., 2020 | Isometric | Ibs | Males:  53.25±14.52  Females:  54.41±14.57 | 0.08 |
| Hughes, et al., 1999 | Isometric | Nm | Males:  Flexed 30°:  AR: 20-29 = 77±21  AR: 30-39 = 64±12  AR: 40-49 = 69±12  AR: 50-59 = 59±12  AR: 60+ = 55±13  Flexed 60°:  AR: 20-29 = 86±20  AR: 30-39 = 71±22  AR: 40-49 = 82±15  AR: 50-59 = 68±11  AR: 60+ = 62±11  Flexed 90°:  AR: 20-29 = 87±21  AR: 30-39 = 78±17  AR: 40-49 = 81±17  AR: 50-59 = 70±16  AR: 60+ = 64±12  Females:  Flexed 30°:  AR: 20-29; 38±15  AR: 30-39; 48±14  AR: 40-49; 36±14  AR: 50-59; 27±10  AR: 60+; 24±9  Flexed 60°:  AR: 20-29; 45±18  AR: 30-39; 54±18  AR: 40-49; 39±14  AR: 50-59; 32±13  AR: 60+; 28±9  Flexed 90°:  AR: 20-29; 46±16  AR: 30-39; 55±20  AR: 40-49; 38±11  AR: 50-59; 36±13  AR: 60+; 27±8 | Extension:  Flexed 30°:  AR: 20-29 = 1.86  AR: 30-39 = 1.33  AR: 40-49 = 2.75  AR: 50-59 = 2.67  AR: 60+ = 2.38  Flexed 60°:  AR: 20-29 = 2.05  AR: 30-39 = 0.77  AR: 40-49 = 2.87  AR: 50-59 = 3.27  AR: 60+ = 3.09  Flexed 90°:  AR: 20-29 = 1.95  AR: 30-39 = 1.35  AR: 40-49 = 2.53  AR: 50-59 = 2.13  AR: 60+ = 3.08 |
| Andrews, et al., 1996 | Isometric | N | Males:  AR: 50-59 = 320.7±54.4  AR: 60-69 = 280.1±56.7  Females:  AR: 50-59 = 180.6±38  AR: 60-69 = 153±34.7 | AR: 50-59 = 2.57  AR: 60-69 = 1.65 |
| Marcondes, et al., 2019 | Isokinetic:  60°/s  180°/s | Percent Body Mass | Males:  60°/s = 126.8±11.6  180°/s = 215.9±19.2  Females:  60°/s = 93.1±9.9  180°/s = 145.1±14.1 | 60°/s = 2.91  180°/s = 3.69 |
| Cahalan, et al., 1989 | Isokinetic:  60°/s  180°/s  300°/s | N, Nm | Males:  N = 77±16.5  60°/s = 88.5±19.5  180°/s = 74±16.5  300°/s = 62±15.5  Females:  N = 38±6.5  60°/s = 40±8  180°/s = 32.5±7  300°/s = 26.5±6 | N = 2.36  60°/s = 2.45  180°/s = 2.52  300°/s = 2.29 |
| Shklar and Dvir, 1995 | Isokinetic:  60°/s  120°/s  180°/s | Nm | Males:  Con. 60°/s = 84.9±20.5  Con. 120°/s = 82.1±21.1  Con. 180°/s = 73.3±18.4  Ecc. 60°/s = 112.2±30  Ecc. 120°/s = 113.5±33.1  Ecc. 180°/s = 113.9±30.2  Females:  Con.60°/s = 38.7±9.1  Con.120°/s = 38±6.9  Con.180°/s = 35.5±7.3  Ecc.60°/s = 56.3±8.2  Ecc.120°/s = 58.1±9.6  Ecc.180°/s = 59.8±8.2 | Con. 60°/s = 2.25  Con. 120°/s = 2.09  Con. 180°/s = 0.11  Ecc. 60°/s = 1.86  Ecc. 120°/s = 1.67  Ecc. 180°/s = 1.79 |
| Khalaf and Parnianpour, 2001 | Isokinetic:  10°/s  50°/s  100°/s  50°/s  200°/s  250°/s | Nm | Males:  10°/s = 92.75±19.22  50°/s = 86.15±13.37  100°/s = 73.28±15.07  150°/s = 66.54±11.61  200°/s = 58.23±11.33  250°/s = 53.32±12.78  Female:  10°/s = 53.97±11.21  50°/s = 43.08±10.03  100°/s = 34.45±9  150°/s = 29.99±10.24  200°/s = 22.64±6.24  250°/s = 20.41±8.76 | 10°/s = 2.02  50°/s = 3.22  100°/s = 2.58  150°/s = 3.15  200°/s = 3.14  250°/s = 2.58 |
| Mayer, et al., 1994 | Isometric; Isokinetic:  Con. 300°/s  Con. 240°/s  Con. 180°/s  Con. 60°/s  Ecc. 60°/s  Ecc. 120°/s  Ecc. 180°/s  Ecc. 240°/s | Nm | Males:  ISO. = 93±24  IKO. Con. 300°/s = 55±15  IKO. Con. 240°/s = 56±14  IKO. Con. 180°/s = 57±12  IKO. Con. 60°/s = 67±16  IKO. Ecc. 60°/s = 78±17  IKO. Ecc. 120°/s = 89±19  IKO. Ecc. 180°/s = 77±10  IKO. Ecc. 240°/s = 74±14  Females:  ISO. = 54±12  IKO. Con. 300°/s = 32±7  IKO. Con. 240°/s = 31±9  IKO. Con. 180°/s = 32±8  IKO. Con. 60°/s = 38±10  IKO. Ecc. 60°/s = 44±9  IKO. Ecc. 120°/s = 55±16  IKO. Ecc. 180°/s = 57±10  IKO. Ecc. 240°/s = 49±6 | ISO. = 1.63  IKO. Con. 300°/s = 1.53  IKO. Con. 240°/s = 1.79  IKO. Con. 180°/s = 2.08  IKO. Con. 60°/s = 1.81  IKO. Ecc. 60°/s = 2  IKO. Ecc. 120°/s = 1.79  IKO. Ecc. 180°/s = 2  IKO. Ecc. 240°/s = 1.88 |
| Danneskiold-Samsoe, et al., 2009 | Isometric;  Isokinetic:  30°/s  60°/s  90°/s  120°/s | N, Nm | Males (Nm):  AR: 20-29 = 72.1±19.5 (60°/s), 66.6±18.2 (90°/s), 64.6±18.5 (120°/s)  AR: 30-39 = 65.6±15.1 (60°/s), 59.6±16.1 (90°/s), 56.9±16.1 (120°/s)  AR: 40-49 = 64.0±15.3 (60°/s), 60.1±12.8 (90°/s), 56.7±13.1 (120°/s)  AR: 50-59 = 58.2±7.2 (60°/s), 55.3±7.6 (90°/s), 47.8±5.2 (120°/s)  AR: 60-69 = 52.6±12.6 (60°/s), 51.1±11.3 (90°/s), 45.6±10.9 (120°/s)  AR: 70-79 = 49.6±8.3 (60°/s), 44.8±6.2 (90°/s), 43.3±5.2 (120°/s)  Males (N):  AR: 20-29 = 91.9±19.9  AR: 30-39 = 78.4±18.4  AR: 40-49 = 79.8±18.4  AR: 50-59 = 80.3±4.6  AR: 60-69 = 77.4±16.4  AR: 70-79 = 69.8±13.5  Females **(**Nm):  AR: 20-29 = 33.6±6.8 (60°/s), 31.3±6.2 (90°/s), 31.8±6.6 (120°/s)  AR: 30-39 = 35.2±6.8 (60°/s), 31.9±8.1 (90°/s), 30.1±7.8 (120°/s)  AR: 40-49 = 34.6±6.7 (60°/s), 31.3±7.4 (90°/s), 30.4±7.0 (120°/s)  AR: 50-59 = 32.2±6.2 (60°/s), 29.7±6.8 (90°/s), 29.3±6.9 (120°/s)  AR: 60-69 = 30.4±6.0 (60°/s), 28.7±5.5 (90°/s), 28.3±6.3 (120°/s)  AR: 70-79 = 27.2±6.1 (60°/s), 25.1±6.4 (90°/s), 23.7±5.4 (120°/s)  Females (N):  AR: 20-29 = 43.5±8.5  AR: 30-39 = 47.7±8.5  AR: 40-49 = 46.3±14.3  AR: 50-59 = 43.7±8.7  AR: 60-69 = 41.7±10.1  AR: 70-79 = 37.7±9.3 | Nm:  AR: 20-29 = 1.97 (60°/s), 1.94 (90°/s), 1.77 (120°/s)  AR: 30-39 = 2.01 (60°/s), 1.72 (90°/s), 1.66 (120°/s)  AR: 40-49 = 1.92 (60°/s), 2.25 (90°/s), 2.01 (120°/s)  AR: 50-59 = 3.61 (60°/s), 3.37 (90°/s), 3.56 (120°/s)  AR: 60-69 = 1.76 (60°/s), 1.98 (90°/s), 1.59 (120°/s)  AR: 70-79 = 2.69 (60°/s), 3.18 (90°/s), 3.77 (120°/s)  N:  AR: 20-29 = 2.43  AR: 30-39 = 1.67  AR: 40-49 = 1.82  AR: 50-59 = 7.76  AR: 60-69 = 2.18  AR: 70-79 = 2.38 |
