# Supplementary material for: Sex differences in strength at the shoulder: a systematic review
Source: PeerJ. 2024 Mar 20;12:e16968. doi: 10.7717/peerj.16968 (PMC10960529; doi:10.7717/peerj.16968)
Supplement: Supplemental Information 8 — Isometric (ISO) and isokinetic (IKO) data of concentric (Con) and Eccentric (Ecc) movement types. Age ranges (AR) included. Outcomes are relative to the described measurement unit; where available, effect sizes were extracted or calculated (Cohen’s d). [file peerj-12-16968-s008.docx]

# **Supplementary Table 7: Extracted data for studies with shoulder horizontal flexion data.**

Isometric (ISO) and isokinetic (IKO) data of concentric (Con) and Eccentric (Ecc) movement types. Age ranges (AR) included. Outcomes are relative to the described measurement unit; where available, effect sizes were extracted or calculated (Cohen's d).

| **Title** | **Movement Type** | **Measurement Unit** | **Outcomes** | **Effect Size (Cohen’s d)** |
| --- | --- | --- | --- | --- |
| VanHarlinger, et al., 2015 | Isometric | Kg | Males:  AR: 20-24 = 17.3±6.5  AR: 25-29 = 22.8±5.6  AR: 30-34 = 22±6.6  AR: 35-39 = 18.4±6.3  AR: 40-44 = 23.6±3  AR: 45-49 = 16.5±8.2  AR: 50-54 = 17.1±5.2  AR: 55-59 = 19.2±6.7  AR: 60-64 = 19.5±4.5  Females:  AR: 20-24 = 9.7±4.4  AR: 25-29 = 8.2±3.4  AR: 30-34 = 8.8±4  AR: 35-39 = 9.1±4  AR: 40-44 = 9.3±4.1  AR: 45-49 = 11.56±4.3  AR: 50-54 = 9.21±2.3  AR: 55-59 = 9.78±3.6  AR: 60-64 = 10.1±3.7 | AR: 20-24 = 1.17  AR: 25-29 = 2.61  AR: 30-34 = 2  AR: 35-39 = 1.48  AR: 40-44 = 4.77  AR: 45-49 = 0.60  AR: 50-54 = 1.52  AR: 55-59 = 1.41  AR: 60-64 = 2.09 |
| Huberman, et al., 2020 | Isometric | Ibs | Males:  41.80±14.90  Females:  43.59±14.95 | 0.12 |
