# Supplementary material for: Sex differences in strength at the shoulder: a systematic review
Source: PeerJ. 2024 Mar 20;12:e16968. doi: 10.7717/peerj.16968 (PMC10960529; doi:10.7717/peerj.16968)
Supplement: Supplemental Information 9 — Isometric (ISO) and isokinetic (IKO) data of concentric (Con) and Eccentric (Ecc) movement types. Age ranges (AR) included. Outcomes are relative to the described measurement unit; where available, effect sizes were extracted or calculated (Cohen’s d). [file peerj-12-16968-s009.docx]

# **Supplementary Table 8: Extracted data for studies with shoulder horizontal extension data.**

Isometric (ISO) and isokinetic (IKO) data of concentric (Con) and Eccentric (Ecc) movement types. Age ranges (AR) included. Outcomes are relative to the described measurement unit; where available, effect sizes were extracted or calculated (Cohen's d).

| **Title** | **Movement Type** | **Measurement Unit** | **Outcomes** | **Effect Size (Cohen’s d)** |
| --- | --- | --- | --- | --- |
| VanHarlinger, et al., 2015 | Isometric | kg | Males:  AR: 20-24 = 11.2±4.1  AR: 25-29 = 12.4±3.9  AR: 30-34 = 12.3±4  AR: 35-39 = 11.2±2.4  AR: 40-44 = 13.7±3  AR: 45-49 = 8.7±3.2  AR: 50-54 = 11.4±5.4  AR: 55-59 = 11.2±2.1  AR: 60-64 = 9.4±2.3  Females:  AR: 20-24 = 5.8±3.3  AR: 25-29 = 4.9±2.8  AR: 30-34 = 4.7±2.4  AR: 35-39 = 5.4±2.7  AR: 40-44 = 4.9±3.2  AR: 45-49 = 6.1±2.9  AR: 50-54 = 4.8±1.9  AR: 55-59 = 5±2  AR: 60-64 = 4.4±1.2 | AR: 20-24 = 1.32  AR: 25-29 = 1.92  AR: 30-34 = 1.90  AR: 35-39 = 2.42  AR: 40-44 = 2.93  AR: 45-49 = 0.81  AR: 50-54 = 1.22  AR: 55-59 = 2.95  AR: 60-64 = 2.17 |
| Huberman, et al., 2020 | Isometric | Ibs | Males:  27.60±8.25  Females:  26.88±8.27 | 0.12 |
