# Supplementary material for: Sex differences in strength at the shoulder: a systematic review
Source: PeerJ. 2024 Mar 20;12:e16968. doi: 10.7717/peerj.16968 (PMC10960529; doi:10.7717/peerj.16968)
Supplement: Supplemental Information 10 — Isometric (ISO) and isokinetic (IKO) data of concentric (Con) and Eccentric (Ecc) movement types. Age ranges (AR) included. Outcomes are relative to the described measurement unit; where available, effect sizes were extracted or calculated (Cohen’s d). [file peerj-12-16968-s010.docx]

# **Supplementary Table 9: Extracted data for studies with shoulder internal rotation data.**

Isometric (ISO) and isokinetic (IKO) data of concentric (Con) and Eccentric (Ecc) movement types. Age ranges (AR) included. Outcomes are relative to the described measurement unit; where available, effect sizes were extracted or calculated (Cohen's d).

| **Title** | **Movement Type** | **Measurement Unit** | **Outcomes** | **Effect Size (Cohen’s d)** |
| --- | --- | --- | --- | --- |
| Roy, et al., 2009 | Isometric | Nm | Males:  Young = 46.8±16.5  Mid = 50.5±16.7  Old = 37.1 ± 10.1  Females:  Young = 23.2±9.0  Mid = 18.8±5.7  Older = 19.8 ± 7.1 | Young = 24.5  Mid = 1.43  Older = 1.89 |
| Murray, et al., 1985 | Isometric | kg-cm | Males:  Young 0° = 592±27  Old 0° = 444±17  Females:  Young 0° = 289±12  Old 0° = 229±15 | Young 0° = 11.22  Old 0° = 24.41 |
| Chezar, et al., 2013 | Isometric | Nm/kg | Males:  AR: 30-39 = 51±13  AR: 40-49 = 55±11  AR: 50-59 = 57±20  AR: 60-69 = 45±16  Females:  AR: 30-39 = 33±10  AR: 40-49 = 37±12  AR: 50-59 = 34±16  AR: 60-69 = 32±9 | AR: 30-39 = 1.38  AR: 40-49 = 1.64  AR: 50-59 = 1.15  AR: 60-69 = 0.813 |
| Riemann, et al., 2010 | Isometric | Percent Body Mass | Males:  Pronated 90° = 21.6±6.3  Neutral Seated = 21.1±5.9  Seated 30° = 21.4±6.0  Females:  Prone 90° = 11.6±5.1  Neutral Seated = 12.4±5.0  Seated 30° = 12.4±4.5 | Pronated 90° = 1.59  Neutral Seated = 8.7  Seated 30° = 1.5 |
| VanHarlinger, et al., 2015 | Isometric | kg | Males:  AR: 20-24 = 10.12±4.3  AR: 25-29 = 11.7±3.7  AR: 30-34 = 14.2±5.9  AR: 35-39 = 10.5±6.6  AR: 40-44 = 15.1±4.3  AR: 45-49 = 11.3±4.9  AR: 50-54 = 8.3±3  AR: 55-59 = 10.7±3.2  AR: 60-64 = 11.3±3.7  Females:  AR: 20-24 = 5.7±2.4  AR: 25-29 = 4.8±2.1  AR: 30-34 = 4.5±2.4  AR: 35-39 = 4.7±2.4  AR: 40-44 = 6.3±2.9  AR: 45-49 = 7±2  AR: 50-54 = 5±1.6  AR: 55-59 = 4.7±2.2  AR: 60-64 = 5.9±3.8 | AR: 20-24 = 1.03  AR: 25-29 = 1.86  AR: 30-34 = 1.64  AR: 35-39 = 0.88  AR: 40-44 = 2.05  AR: 45-49 = 0.88  AR: 50-54 = 1.1  AR: 55-59 = 1.88  AR: 60-64 = 1.46 |
| McKay, et al., 2017 | Isometric | N | Males:  AR: 20-59 = 202.4±55.9  AR: 60+ = 159.7±42.9  Females:  AR: 20-59 = 109.7±33.6  AR: 60+ = 86±27.5 | AR: 20-59 = 1.66  AR: 60+ = 1.72 |
| Huberman, et al., 2020 | Isometric | Ibs | Males:  30.63±10.78  Females:  32.18±10.81 | 0.14 |
| Westrick, et al., 2013 | Isometric | N/kg | Males:  0.27±0.06  Females:  0.21±0.05 | 0.83 |
| Hughes, et al., 1999 | Isometric | Nm | Males:  Internal Rotation (0°) Abduction (15°):  AR: 20-29 = 51±19  AR: 30-39 = 48±10  AR: 40-49 = 48±6  AR: 50-59 = 43±9  AR: 60+ = 40±7  Internal Rotation (0°) Abduction (15°):  AR: 20-29 = 53±19  AR: 30-39 = 49±9  AR: 40-49 = 51±9  AR: 50-59 = 41±7  AR: 60+ = 42±7  External Rotation (30°) Abduction (90°):  AR: 20-29 = 48±12  AR: 30-39 = 44±7  AR: 40-49 = 41±12  AR: 50-59 = 37±7  AR: 60+ = 35±5  External Rotation (60°) Abduction (90°):  AR: 20-29 = 48±12  AR: 30-39 = 44±7  AR: 40-49 = 41±12  AR: 50-59 = 37±7  AR: 60+ = 35±5  Females:  Internal Rotation (0°) Abduction (15°):  AR: 20-29 = 28±10  AR: 30-39 = 31±8  AR: 40-49 = 27±6  AR: 50-59 = 20±4  AR: 60+ = 16±5  Internal Rotation (0°) Abduction (15°):  AR: 20-29 = 28±10  AR: 30-39 = 33±10  AR: 40-49 = 27±8  AR: 50-59 = 20±5  AR: 60+ = 16±6  External Rotation (30°) Abduction (90°):  AR: 20-29 = 27±11  AR: 30-39 = 28±8  AR: 40-49 = 22±7  AR: 50-59 = 17±6  AR: 60+ = 13±5  External Rotation (60°) Abduction (90°):  AR: 20-29 = 24±11  AR: 30-39 = 25±8  AR: 40-49 = 18±7  AR: 50-59 = 15±5  AR: 60+ = 12±5 | Internal Rotation (0°) Abduction (15°):  AR: 20-29 = 1.21  AR: 30-39 = 1.7  AR: 40-49 = 3.5  AR: 50-59 = 2.56  AR: 60+ = 3.43  Internal Rotation (0°) Abduction (15°):  AR: 20-29 = 1.32  AR: 30-39 = 1.78  AR: 40-49 = 2.67  AR: 50-59 = 3  AR: 60+ = 3.71  External Rotation (30°) Abduction (90°):  AR: 20-29 = 1.75  AR: 30-39 = 2.29  AR: 40-49 = 1.58  AR: 50-59 = 2.86  AR: 60+ = 4.4  External Rotation (60°) Abduction (90°):  AR: 20-29 = 2  AR: 30-39 = 2.71  AR: 40-49 = 1.92  AR: 50-59 = 3.14  AR: 60+ = 4.6 |
| Magnusson, et al., 1995 | Isometric | Nm/kg | Males:  Left = 0.59±0.05  Right = 0.55±0.04  Females:  Left = 0.36±0.04  Right = 0.39±0.03 | Left = 4.6  Right = 4 |
| Stausholm, et al., 2021 | Isometric | Nm/kg | Males:  1.57±0.30  Females:  1.31±0.31 | 0.87 |
| Pontillo and Sennet, 2020 | Isometric | kg | Males:  12±3.8  Females:  8.7±2.6 | 0.87 |
| Andrews, et al., 1996 | Isometric | N | Males:  AR: 50-59 = 193.3±40.2  AR: 60-69 = 163.3±28.9  Females:  AR: 50-59 = 100.7±19.9  AR: 60-69 = 92.3±18.7 | AR: 50-59 = 2.30  AR: 60-69 = 2.57 |
| Alizadehkhaiyat, et al., 2014 | Isometric | N | Males:  157.6±40.1  Females:  95.1±18.3 | 1.56 |
| Cools, et al., 2016 | Isometric | N | Males:  90-0° = 165.4±29.6  90-90° = 183.3±45.8  Females:  90-0° = 112.5±20.4  90-90° = 114.0±31.3 | 90-0° = 1.79  90-90° = 1.51 |
| Kramer and Ng, 1995 | Isometric | Nm | Males:  Isokinetic Dynamometer = 58±19  Hand-Held Dynamometer = 41±7  Females:  Isokinetic Dynamometer = 22±7  Hand-Held Dynamometer = 23±5 | Isokinetic Dynamometer = 1.89  Hand-Held Dynamometer = 2.57 |
| Marcondes, et al., 2019 | Isokinetic:  60°/s  180°/s | Percent Body Mass | Males:  60°/s = 81.4±8.6  180°/s = 161.5±12.5  Females:  60°/s = 67.5±4.7  180°/s = 124.3±12.1 | 60°/s = 1.62  180°/s = 2.98 |
| Cahalan, et al., 1989 | Isokinetic:  60°/s  180°/s  300°/s | N, Nm | Males:  N = 57±17  60 °/s = 44.5±13.5  180 °/s = 38.5±12.5  300 °/s = 33±12  Females:  N = 26.5±4.5  60 °/s = 21±4  180 °/s = 16.5±4  300 °/s = 13±4 | N = 1.79  60 °/s = 1.74  180 °/s = 1.76  300 °/s = 1.67 |
| Shklar and Dvir, 1995 | Isokinetic:  60°/s  120°/s  180°/s | Nm | Males:  Con.60° = 42.6±13.4  Con.120° = 38.2±11.9  Con.180° = 37.1±11.4  Ecc.60° = 47.4±14.8  Ecc.120° = 46.5±15.1  Ecc.180° = 45.2±15.9  Females:  Con.60° = 22.6±3.4  Con.120° = 21.2±2.6  Con.180° = 20.1±3.2  Ecc.60° = 27.4±6.2  Ecc.120° = 26.1±6.4  Ecc.180° = 26.3±6.6 | Con.60° = 1.49  Con.120° = 1.43  Con.180° = 1.49  Ecc.60° = 1.35  Ecc.120° = 1.35  Ecc.180° = 1.19 |
| McMaster, et al., 1992 | Isokinetic:  30°/s  180°/s | Foot-pounds | Males:  Con.30°.L = 36.8±10.4  Con.30°.R = 39.3±9.6  Con.180°.L = 41.8±9.4  Con.180°.R = 37.6±12.1  Females:  Con.30°.L = 26.7±4.4  Con.30°.R = 27.2±3.7  Con.180°.L = 27.6±4.4  Con.180°.R = 27.9±3.8 | Con.30°.L = 0.97  Con.30°.R = 1.26  Con.180°.L = 1.51  Con.180°.R = 1.63 |
| Ivey, et al., 1985 | Isokinetic:  60°/s | Foot-Pounds | Males:  Slow = 36.4±12.2  Fast = 32.7±11.0  Females:  Slow = 19.6±2.9  Fast = 17.1±3.0 | Slow = 1.38  Fast = 1.42 |
| Reid, et al., 1989 | Isokinetic:  60°/s | Nm | Males:  Lying = 40±16  Standing = 42±19  Females:  Lying = 19±5  Standing = 23±5 | Lying = 1.31  Standing = 1 |
| Motta, et al., 2019 | Isokinetic:  60°/s  240°/s | Nm/kg | Males:  Con.60° = 160.30±23.55  Con.240° = 107.20±19.00  Ecc.240° = 232.50±34.65  Females:  Con.60° = 128.55±19.90  Con.240° = 86.12±16.45  Ecc.240° = 196.10±34.60 | Con.60° = 1.35  Con.240° = 1.11  Ecc.240° = 1.05 |
| Maddux, et al., 1989 | Isokinetic:  60°/s  180°/s | Foot-Pounds | Males:  60°/s = 32±10  180°/s = 30±8  Females:  60°/s = 17±4  180°/s = 13±4 | 60°/s = 1.5  180°/s = 2.13 |
| Hartsell, 1998 | Isokinetic  60°/s  120°/s  180°/s | Nm | Males:  Con.Sit:  60°/s = 86.35±23.09  120°/s = 83.82±23.50  78.11±23.12 (180 °/s)  Con.Stand:  60 °/s = 81.98±20.4  120°/s = 76.30±25.47  180 °/s = 70.24±23.12  Ecc.Sit:  60 °/s = -94.22±26.28  120°/s =-93.55±30.53  180 °/s = -92.37±31.53  Ecc.Stand:  60 °/s = -90.54±22.97  120°/s =-85.84±26.15  180 °/s = -84.66±24.38  Females:  Con.Sit:  60 °/s = 45.91±6.62  120°/s = 41.22±6.67  180 °/s = 37.73±7.48  Con.Stand:  60 °/s = 47.11±10.56  120°/s = 42.42±7.82  180 °/s = 39.06±8.07  Ecc.Sit:  60 °/s = 52.81±7.87  120°/s = -51.30±8.90  180 °/s = -50.97±8.58  Ecc. Stand:  60 °/s = -52.65±13.38  120°/s = -51.97±10.96  180 °/s = -56.17±3.08 | Con.Sit:  60°/s = 1.75  120°/s =1.81  180 °/s = 1.75  Con.Stand:  60°/s = 1.71  120°/s = 1.33  180 °/s = 1.35  Ecc.Sit:  60°/s = 1.58  120°/s = 1.38  180 °/s = 1.31  Ecc. Stand:  60 °/s = 1.65  120°/s = 1.29  180 °/s = 1.17 |
| VanMeeteren, et al., 2002 | Isokinetic:  60 °/s  120°/s  180°/s | Nm | Males:  49.25±13.25  Females:  25.5±6.95 | 1.79 |
| Hill, et al., 2005 | Isokinetic:  60°/s  90°/s  120°/s | Nm | Males:  Sitting (60°/s):  Left = 44.1±14.5  Right = 47.6±14.1  Sitting (90°/s):  Left = 41.7±13.4  Right = 46±12  Sitting (120°/s):  Left = 43±12.1  Right = 44.7±12.5  Lying (60°/s):  Left = 43.8±13.7  Right = 47.5±14.2  Lying (90°/s):  Left = 38.6±10  Right = 41.5±12.3  Lying (120°/s):  Left = 37.4±7.9  Right = 38.4±10.6  Females:  Sitting (60 °/s):  Left = 27.3±4.5  Right = 26.7±3.4  Sitting (90 °/s):  Left = 26±5.2  Right = 26.7±3.9  Sitting (120 °/s):  Left = 24±3.7  Right = 25.3±4.9  Lying (60 °/s):  Left = 27±2.8  Right = 26.7±4.0  Lying (90 °/s):  Left = 24.3±3.3  Right = 24.8±3.1  Lying (120 °/s):  Left = 23.3±2.9  Right = 23.3±2.7 | Sitting (60 °/s):  Left = 1.16  Right = 1.48  Sitting (90 °/s):  Left = 1.17  Right = 1.61  Sitting (120 °/s):  Left = 1.57  Right = 1.55  Lying (60 °/s):  Left = 1.23  Right = 1.46  Lying (90 °/s):  Left = 1.43  Right = 1.36  Lying (120 °/s):  Left = 1.78  Right = 1.42 |
| VanCingel, et al., 2007 | Isokinetic:  60°/s  120°/s | Nm/kg | Males:  Con.60°/s = 0.69±0.11  Con.120°/s = 0.63±0.11  Females:  Con.60°/s = 0.45±0.09  Con.120°/s = 0.42±0.09 | Con.60°/s = 2.18  Con.120°/s = 1.91 |
| Murgia, et al., 2018 | Isokinetic:  60°/s  90°/s | Nm | Males:  Young 60°/s = 0.65±0.20  Young 90°/s = 0.65±0.24  Old 60°/s = 0.55±0.12  Old 90°/s = 0.56±0.11  Females:  Young 60°/s = 0.35±0.06  Young 90°/s = 0.36±0.05  Old 60°/s = 0.33±0.12  Old 90°/s = 0.35±0.12 | Young 60°/s = 1.5  Young 90°/s = 1.21  Old 60°/s = 1.83  Old 90°/s = 1.91 |
| Barrenetxea-Garcia, et al., 2019 | Isokinetic:  60°/s  240°/s | Nm | Males:  60°/s = 50.41±9.82  240°/s = 42.88±7.43  Females:  60°/s = 30.50±6.62  240°/s = 24.10±5.26 | 60°/s = 2.03  240°/s = 2.53 |
| Ellenbecker and Roetert, 2003 | Isokinetic  210°/s  300°/s | Nm/kg | Males:  210°/s = 55.1±14.6  300°/s = 46.5±13.2  Females:  210°/s = 33.2±8.6  300°/s = 28.8±9/4 | 210°/s = 1.5  300°/s = 1.34 |
| Mayer, et al., 1994 | Isometric; Isokinetic:  300°/s  240°/s  180°/s  60°/s  -60°/s  -120°/s  -180°/s  -240°/s | Nm | Males:  ISO = 43±12  IKO.Con.300° = 34±8  IKO.Con.240° = 35±8  IKO.Con.180° = 37±8  IKO.Con.60° = 42±8  IKO.Ecc.60° = 46±13  IKO.Ecc.120° = 41±10  IKO.Ecc.180° = 42±6  IKO.Ecc.240° = 43±8  Females:  ISO = 21±7  IKO.Con.300° = 19±3  IKO.Con.240° = 18±5  IKO.Con.180° = 20±4  IKO.Con.60° = 23±5  IKO.Ecc.60° = 25±7  IKO.Ecc.120° = 23±6  IKO.Ecc.180° = 27±4  IKO.Ecc.240° = 24±3 | ISO = 1.83  IKO.Con.300° = 1.88  IKO.Con.240° = 2.12  IKO.Con.180° = 2.12  IKO.Con.60° = 2.38  IKO.Ecc.60° = 1.62 IKO.Ecc.120° = 1.8 IKO.Ecc.180° = 2.5 IKO.Ecc.240° = 2.38 |
| Smith, et al., 2001 | Isometric; Isokinetic:  90 °/s | Nm | Males:  ISO = 52.8±9.9  IKO = 47.5±7.3  Females:  ISO = 25.9±5.5  IKO = 25.4±3.8 | ISO = 2.71  IKO = 3.03 |
| Hageman, et al., 1989 | Isokinetic:  60°/s  180°/s | Nm | Males:  Con.Flexion.60°/s = 48±10.9  Con.Abduction.60°/s = 48.6±10.8  Ecc.Flexion.60°/s = 51.3±11.1  Ecc.Abduction.60°/s = 54.9±13.8  Con.Flexion.180°/s = 42.5±9.3  Con.Abduction.180°/s = 42.3±8.3  Ecc.Flexion.180°/s = 49.1±10.6  Ecc.Abduction.180°/s = 54.1±12.9  Females:  Con.Flexion.60°/s = 22.1±5.2  Con.Abduction.60°/s = 23.7±5.1  Ecc.Flexion.60°/s = 24.9±6.7  Ecc.Abduction.60°/s = 29.2±7.4  Con.Flexion.180°/s = 21.9±7.4  Con.Abduction.180°/s = 22.2±5.6  Ecc.Flexion.180°/s = 28.2±10.2  Ecc.Abduction.180°/s = 28.6±8 | Con.Flexion.60°/s = 2.38  Con.Abduction.60°/s = 2.31  Ecc.Flexion.60°/s = 2.38  Ecc.Abduction.60°/s = 1.86  Con.Flexion.180°/s = 2.22  Con.Abduction.180°/s = 2.42  Ecc.Flexion.180°/s = 1.97  Ecc.Abduction.180°/s = 1.98 |
| Aydin, et al., 2000 | Isometric; Isokinetic:  90°/s | Nm, W | Males:  Concentric:  Nm = 49.3±12.3  W = 46.8±12.0  Isometric Preload:  Nm = 56.0±12.2  W = 46.8±12.0  Eccentric:  Nm = 59.5±12.5  W = 63.9±14.2  Females:  Concentric:  Nm = 21.7±3.5  W = 19.0±4.4  Isometric Preload:  Nm = 24.6±3.0  W = 21.4±4.6  Eccentric:  Nm = 28.5±3.1  W = 25.7±4.8 | Concentric:  Nm = 2.24  W = 2.32  Isometric:  Nm = 2.57  W = 2.17  Eccentric:  Nm = 2.48  W = 2.69 |
| Kramer and Ng, 1996 | Isometric; Isokinetic:  0°/s  60°/s  120°/s | Nm | Males:  ISO = 58±19  IKO.Con.60°/s = 44±14  IKO.Con.120°/s = 43±15  IKO.Ecc.60°/s = 54±20  IKO.Ecc.120°/s = 57±17  Females:  ISO = 27±7  IKO.Con.60°/s = 23±5  IKO.Con.120°/s = 20±6  IKO.Ecc.60°/s = 27±6  IKO.Ecc.120°/s = 27±6 | ISO = 1.63  IKO.Con.60°/s = 1.5  IKO.Con.120°/s = 1.53  IKO.Ecc.60°/s = 1.35  IKO.Ecc.120°/s = 1.76 |
