# Supplementary material for: Sex differences in strength at the shoulder: a systematic review
Source: PeerJ. 2024 Mar 20;12:e16968. doi: 10.7717/peerj.16968 (PMC10960529; doi:10.7717/peerj.16968)
Supplement: Supplemental Information 11 — Isometric (ISO) and isokinetic (IKO) data of concentric (Con) and Eccentric (Ecc) movement types. Age ranges (AR) included. Outcomes are relative to the described measurement unit; where available, effect sizes were extracted or calculated (Cohen’s d). [file peerj-12-16968-s011.docx]

# **Supplementary Table 10: Extracted data for studies with shoulder external rotation data.**

Isometric (ISO) and isokinetic (IKO) data of concentric (Con) and Eccentric (Ecc) movement types. Age ranges (AR) included. Outcomes are relative to the described measurement unit; where available, effect sizes were extracted or calculated (Cohen's d).

| **Title** | **Movement Type** | **Measurement Unit** | **Outcomes** | **Effect Size (Cohen’s d)** |
| --- | --- | --- | --- | --- |
| Kim, et al., 2009 | Isometric | Nm | Males:  AR:40-49 = 120.6±33.3  AR:50-59 = 112.8±22.6  AR:60-69 = 99.0±24.6  Females:  AR:40-49 = 62.8±15.7  AR:50-59 = 65.7±14.7  AR:60-69 = 61.8±12.8 | AR: 40-49 = 1.74  AR: 50-59 = 2.08  AR: 60-69 = 1.51 |
| Murray, et al., 1985 | Isometric | kg-cm | Males:  Young 0° = 335±15  Old 0° = 280±10  Females:  Young0° = 186±8  Old 0° = 152±11 | Young 0° = 9.93  Old 0° = 12.8 |
| Roy, et al., 2009 | Isometric | Nm | Males:  Young = 33.6±9.0  Mid = 40.0±11.2  Older = 25.9 ± 11.2  Females:  Young = 17.2±4.8  Mid = 17.5±3.7  Older = 14.8 ± 5.9 | Young = 1.47  Mid = 1.82  Older = 2.01 |
| Chezar, et al., 2013 | Isometric | Nm/kg | Males:  AR: 30-39 = 102±35  AR: 40-49 = 110±35  AR: 50-59 = 122±43  AR: 60-69 = 89±35  Females:  AR: 30-39 = 44±12  AR: 40-49 = 47±18  AR: 50-59 = 47±18  AR: 60-69 = 42±12 | AR: 30-39 = 1.66  AR: 40-49 = 1.80  AR: 50-59 = 1.74  AR: 60-69 = 1.34 |
| Lannersten, et al., 1993 | Isometric | Nm | Males:  AR: 19-34 = 27±9.1  AR: 35-44 = 28.1±10.6  AR: 45-65 = 25.1±6.9  Females:  AR: 19-34 = 15.1±3.8  AR: 35-44 = 14.9±3.2  AR: 45-65 = 14.8±3.2 | AR: 19-34 = 0.14  AR: 35-44 = 1.25  AR: 45-65 = 1.49 |
| Riemann, et al., 2010 | Isometric | Percent Body Mass | Males:  Prone.90° = 18.7±5.9  Neutral.Seat = 17.8±5.2  Seat.30° = 17.8±5.5  Females:  Prone.90° = 10.8±4.5  Neutral.Seat = 9.9±3.6  Seat.30° = 9.9±3.9 | Prone.90° = 1.34  Neutral.Seat = 1.52  Seat.30° = 1.44 |
| VanHarlinger, et al., 2015 | Isometric | kg | Males:  AR 20-24 = 10±3.1  AR 25-29 = 12.7±2.1  AR 30-34 = 12.4±4  AR 35-39 = 10.1±2.9  AR 40-44 = 12±2.6  AR 45-49 = 10.2±3.6  AR 50-54 = 9±4.1  AR 55-59 = 11.2±3.3  AR 60-64 = 8.4±2.4  Females:  AR 20-24 = 6.3±2  AR 25-29 = 6±2.3  AR 30-34 = 5.3±2.4  AR 35-39 = 5.7±2.4  AR 40-44 = 5.9±2.7  AR 45-49 = 7.7±2.8  AR 50-54 = 6.1±2.2  AR 55-59 = 5.3±1.8  AR 60-64 = 5.6±2.4 | AR 20-24 = 1.19  AR 25-29 = 3.19  AR 30-34 = 1.78  AR 35-39 = 1.52  AR 40-44 = 2.35  AR 45-49 = 0.69  AR 50-54 = 2.90  AR 55-59 = 1.79  AR 60-64 = 1.17 |
| McKay, et al., 2017 | Isometric | N | Males:  AR:20-59 = 134.7±39.6  AR:60+ = 96.7±25.3  Females:  AR:20-59 = 82.2±20.9  AR:60+ = 63.3±19.2 | AR: 20-59 = 1.33  AR: 60+ = 1.32 |
| Huberman, et al., 2020 | Isometric | Ibs | Males:  31.23±9.66  Females:  32.28±9.69 | 0.12 |
| Westrick, et al., 2013 | Isometric | N/kg | Males:  0.20±0.04  Females:  0.16±0.04 | 1 |
| Hughes, et al., 1999 | Isometric | Nm | Males:  Internal Rotation (0°) Abduction (15°):  AR: 20-29 = 31±10  AR: 30-39 = 28±6  AR: 40-49 = 25±6  AR: 50-59 = 24±5  AR: 60+ = 20±5  Internal Rotation (30°) Abduction (15°):  AR: 20-29 = 34±9  AR: 30-39 = 33±9  AR: 40-49 = 32±10  AR: 50-59 = 29±6  AR: 60+ = 26±5  Internal Rotation (0°) Abduction (90°):  AR: 20-29 = 34±8  AR: 30-39 = 31±6  AR: 40-49 = 31±9  AR: 50-59 = 28±6  AR: 60+ = 25±7  External Rotation (60°) Abduction (90°):  AR: 20-29 = 30±6  AR: 30-39 = 30±6  AR: 40-49 = 27±9  AR: 50-59 = 25±5  AR: 60+ = 25±7  Females:  Internal Rotation (0°) Abduction (15°):  AR: 20-29 = 15±6  AR: 30-39 = 19±4  AR: 40-49 = 17±6  AR: 50-59 = 11±3  AR: 60+ = 10±5  Internal Rotation (30°) Abduction (15°):  AR: 20-29 = 18±5  AR: 30-39 = 21±5  AR: 40-49 = 19±5  AR: 50-59 = 14±2  AR: 60+ = 11±5  Internal Rotation (0°) Abduction (90°):  AR: 20-29 = 20±6  AR: 30-39 = 20±6  AR: 40-49 = 20±4  AR: 50-59 = 15±4  AR: 60+ = 12±5  External Rotation (60°) Abduction (90°):  AR: 20-29 = 18±5  AR: 30-39 = 18±4  AR: 40-49 = 17±4  AR: 50-59 = 14±3  AR: 60+ = 12±4 | Internal Rotation (0°) Abduction (15°):  AR: 20-29 = 1.60  AR: 30-39 = 1.5  AR: 40-49 = 1.33  AR: 50-59 = 2.6  AR: 60+ = 1  Internal Rotation (30°) Abduction (15°):  AR: 20-29 = 1.78  AR: 30-39 = 1.33  AR: 40-49 = 1.30  AR: 50-59 = 2.50  AR: 60+ = 3  Internal Rotation (0°) Abduction (90°):  AR: 20-29 = 1.75  AR: 30-39 = 1.83  AR: 40-49 = 1.22  AR: 50-59 = 2.17  AR: 60+ = 1.86  External Rotation (60°) Abduction (90°):  AR: 20-29 = 2  AR: 30-39 = 2  AR: 40-49 = 1.11  AR: 50-59 = 2.2  AR: 60+ = 1.86 |
| Magnusson, et al., 1995 | Isometric | Nm/kg | Males:  Left = 0.53±0.03  Right = 0.52±0.03  Females:  Left = 0.37±0.02  Right = 0.41±0.02 | Left = 3.2  Right = 3.67 |
| Stausholm, et al., 2021 | Isometric | Nm/kg | Males:  1.23±0.31  Females:  1.11±0.5 | 0.39 |
| Guirelli, et al., 2021 | Isometric | N/kg | Males:  1.26±0.22  Females:  1.26±0.22 | 1.20 |
| Pontillo and Sennet, 2020 | Isometric | kg | Males:  10.7±3.4  Females:  7.9±2.5 | 0.82 |
| Andrews, et al., 1996 | Isometric | N | Males:  AR: 50-59 = 155.9±33.1  AR: 60-69 = 139.2±27.2  Females:  AR: 50-59 = 100.4±20.3  AR: 60-69 = 88.4±18.9 | AR: 50-59 = 1.68  AR: 60-69 = 1.87 |
| Alizadehkhaiyat, et al., 2014 | Isometric | N | Males:  114.6±31.6  Females:  73.7±15.8 | 1.29 |
| Cools, et al., 2016 | Isometric | N | Males:  90-0° = 145.2±28.3  90-90° = 106.2±19.8  Females:  90-0° = 113.8±25.3  90-90° = 94.2±23.2 | 90-0° = 1.11  90-90° = 0.61 |
| Kramer and Ng, 1995 | Isometric | Nm | Males:  Isokinetic Dynamometer = 41±11  Hand-Held Dynamometer = 41±7  Females:  Isokinetic Dynamometer = 22±7  Hand-Held Dynamometer = 23±5 | Isokinetic Dynamometer = 1.89  Hand-Held Dynamometer = 2.57 |
| Marcondes, et al., 2019 | Isokinetic;  60 °/s;  180 °/s | Percent Body Mass | Males:  60°/s = 40.1±6.5  180°/s = 69.2±7.8  Females:  60°/s = 33.3±3.3  180°/s = 55.5±5.3 | 60°/s = 1.05  180°/s = 1.76 |
| Cahalan, et al., 1989 | Isokinetic;  60 °/s; 180 °/s; 300 °/s | N, Nm | Males:  N = 32±6.5  60°/s = 26±5.5  180°/s = 19.5±4  300°/s = 14.5±4  Females:  N = 17.5±2.5  60°/s = 13.5±3  180°/s = 7±3  300°/s = 4±2 | N = 2.23  60°/s = 2.27  180°/s = 3.13  300°/s = 2.63 |
| Shklar and Dvir, 1995 | Isokinetic;  60 °/s; 120 °/s; 180 °/s | Nm | Males:  Con.60° = 25.6±7.2  Con.120° = 22.9±6.4  Con.180° = 21.2±5.7  Ecc.60° = 32±8.1  Ecc.120° = 30.1±8.5  Ecc.180° = 31.3±7.9  Females:  Con.60° = 16.3±2.5  Con.120° = 14.1±2.6  Con.180° = 13.5±3.3  Ecc.60° = 19.9±4.6  Ecc.120° = 19.8±4.6  Ecc.180° = 19.6±4.4 | Con.60° = 1.29  Con.120° = 1.38  Con.180° = 1.35  Ecc.60° = 1.49  Ecc.120° = 1.21  Ecc.180° = 1.48 |
| Ivey, et al., 1985 | Isokinetic:  60 °/s  180 °/s | Foot-Pounds | Males:  Slow = 23.8±5.8  Fast = 21.1±6.8  Females:  Slow = 13.9±2.3  Fast = 11.2±2.3 | Slow = 1.71  Fast = 1.46 |
| Reid, et al., 1989 | Isokinetic:  60 °/s | Nm | Males:  Lying = 31±10  Standing = 29±13  Females:  Lying = 16±5  Standing = 13±3 | Lying = 1.5 Standing = 1.23 |
| McMaster, et al., 1992 | Isokinetic:  30°/s  180°/s | Foot-Pounds | Males:  Con.30°.Left = 28.1±6.7  Con.30°.Right = 29.1±6  Con.180°.Left = 25.4±8  Con.180°.Right = 27.4±6.4  Females:  Con.30°.Left = 20.1±3.8  Con.30°.Right = 20.3±3.5  Con.180°.Left = 18.2±3  Con.180°.Right = 16.5±2.3 | Con.30°.Left = 1.19  Con.30°.Right = 1.47  Con.180°.Left = 0.9  Con.180°.Right = 1.70 |
| Motta, et al., 2019 | Isokinetic:  60°/s  240°/s | Nm/kg | Males:  Con.60° = 306.00±59.00  Con.240° = 180.55±29.40  Ecc.240° = 346.80±102.55  Females:  Con.60° = 251.20±37.90  Con.240° = 147.55±19.35  Ecc.240° = 316.35±74.00 | Con.60° = 0.93  Con.240° = 1.12  Ecc.240° = 0.30 |
| Maddux, et al., 1989 | Isokinetic:  60°/s  180°/s | Foot-Pounds | Males:  60°/s = 26±6  180°/s = 18±4  Females:  60°/s = 11±2  180°/s = 8±2 | 60°/s = 2.5  180°/s = 2.5 |
| Hartsell, 1998 | Isokinetic:  60°/s  120°/s  180°/s | Nm | Males:  Con.Sit:  60°/s = 42.42±11.57  120°/s = 33.36±10.05  180°/s = 28.51±9.52  Con. Stand:  60°/s = 45.60±13.30  120°/s = 39.40±12.04  180°/s = 33.52±12.49  Ecc. Sit:  60°/s = -49.45±12.52  120°/s = -46.27±10.63  180°/s = -45.76±12.52  Ecc. Stand:  60°/s = -58.34±11.90  120°/s = -59.01±12.90  180°/s = -60.19±11.95  Females:  Con. Sit:  60°/s = 17.78±3.11  120°/s = 13.91±2.23  180°/s = 14.08±2.06  Con. Stand:  60°/s = 22.63±4.13  120°/s = 16.77±3.14  180°/s = 14.76±2.61  Ecc. Sit:  60°/s = -25.81±5.58  120°/s = -26.66±8.29  180°/s = -26.32±5.72  Ecc. Stand:  60°/s = -30.34±4.97  120°/s = -31.02±5.76  180°/s = -31.52±6.35 | Con.Sit:  60°/s = 2.13  120°/s = 1.94  180°/s = 1.52  Con. Stand:  60°/s = 1.73  120°/s = 1.88  180°/s = 1.50  Ecc. Sit:  60°/s = 1.89  120°/s = 1.85  180°/s = 1.55  Ecc. Stand:  60°/s = 2.35  120°/s = 2.17  180°/s = 2.39 |
| VanMeeteren, et al., 2002 | Isokinetic:  60°/s  120°/s  180°/s | Nm | Males:  29.4±6.45  Females:  16.45±3.45 | 2.01 |
| Hill, et al., 2005 | Isokinetic:  60°/s  90°/s  120°/s | Nm | Males:  Sitting (60 °/s):  Left = 24.8±7.5  Right = 25.3±8.8  Sitting (90 °/s):  Left = 21.5±7.7  Right = 22.5±7.1  Sitting (120 °/s):  Left = 19.2±8.2  Right = 19±6.7  Lying (60 °/s):  Left = 33.9±11.8  Right = 35.6±10.6  Lying (90 °/s):  Left = 32.5±9.9  Right = 33.6±10.1  Lying (120 °/s):  Left = 30.9±8.8  Right = 31.2±9.3  Females:  Sitting (60 °/s):  Left = 14.8±2.3  Right = 12.2±2.3  Sitting (90 °/s):  Left = 12.8±1.9  Right = 12±1.6  Sitting (120 °/s):  Left = 11.7±1.5  Right = 10.5±1.2  Lying (60 °/s):  Left = 22.8±3.0  Right = 20.7±27  Lying (90 °/s):  Left = 21.3±3.4  Right = 19.2±1.5  Lying (120 °/s):  Left = 19.8±2.7  Right = 18.5±1.1 | Sitting (60 °/s):  Left = 1.33  Right = 1.49  Sitting (90 °/s):  Left = 1.13  Right = 1.48  Sitting (120 °/s):  Left = 0.91  Right = 1.27  Lying (60 °/s):  Left = 0.94  Right = 1.41  Lying (90 °/s):  Left = 1.13  Right = 1.43  Lying (120 °/s):  Left = 1.26  Right = 1.37 |
| Hageman, et al., 1989 | Isokinetic:  60 °/s  180 °/s | Nm | Males:  Con.Flexion.60°/s=25.4±7.7  Con.Abduction.60°/s=30.6±5.3  Ecc.Flexion.60°/s=28.1±6.9  Ecc.Abduction.60°/s=34.1±6.9  Con.Flexion.180°/s=25±6.9  Con.Abduction.180°/s=26.7±7.1  Ecc.Flexion.180°/s=30.2±6.9  Ecc.Abduction.180°/s=33.6±6.7  Females:  Con.Flexion.60°/s=13.9±4.5  Con.Abduction.60°/s=16.3±3.3  Ecc.Flexion.60°/s=15.2±4.1  Ecc.Abduction.60°/s=18.2±2.9  Con.Flexion.180°/s=12.3±3.3  Con.Abduction.180°/s=15.4±3.1  Ecc.Flexion.180°/s=17.6±4.3  Ecc.Abduction.180°/s=19.9±4.3 | Con.Flexion.60°/s = 1.49  Con.Abduction.60°/s = 2.69  Ecc.Flexion.60°/s = 1.87  Ecc.Abduction.60°/s = 2.30  Con.Flexion.180°/s = 1.84  Con.Abduction.180°/s = 1.59  Ecc.Flexion.180°/s = 1.83  Ecc.Abduction.180°/s = 2.04 |
| VanCingel, et al., 2007 | Isokinetic:  60 °/s  120 °/s | Nm/kg | Males:  Con.60°/s = 0.49±0.08  Con.120°/s = 0.45±0.08  Ecc.60°/s = 0.63±0.09  Ecc.120°/s = 0.61±0.07  Females:  Con.60°/s = 0.35±0.06  Con.120°/s = 0.34±0.07  Ecc.60°/s = 0.52±0.10  Ecc.120°/s = 0.54±0.09 | Con.60°/s = 1.75  Con.120°/s = 1.38  Ecc.60°/s = 1.22  Ecc.120°/s = 1 |
| Murgia, et al., 2018 | Isokinetic:  60°/s  90°/s | Nm | Males:  Young 60°/s = 0.50±0.06  Young 90°/s = 0.52±0.04  Old 60°/s = 0.39±0.10  Old 90°/s = 0.43±0.13  Females:  Young 60°/s = 0.39±0.10  Young 90°/s = 0.43±0.13  Old 60°/s = 0.28±0.09  Old 90°/s = 0.28±0.08 | Young 60°/s = 1.83  Young 90°/s = 2.25  Old 60°/s = 1.1  Old 90°/s = 1.54 |
| Barrenetxea-Garcia, et al., 2019 | Isokinetic:  60°/s  240°/s | Nm | Males:  60 °/s = 38.59±6.42  240 °/s = 28.47±5.62  Females:  60 °/s = 23.30±5.06  240 °/s = 17.30±2.21 | 60 °/s = 2.38  240 °/s = 1.98 |
| Ellenbecker and Roetert, 2003 | Isokinetic:  210°/s  300°/s | Nm/kg | Males:  210°/s = 37.8±9.1  300 °/s = 32.9±8.1  Females:  210°/s = 24.7±6.1  300 °/s = 21.2±7.3 | 210°/s = 1.44  300 °/s = 1.44 |
| Mayer, et al., 1994 | Isometric; Isokinetic:  300°/s  240°/s  180°/s  60°/s  -60°/s  -120°/s  -180°/s  -240°/s | Nm | Males:  ISO = 30±10  IKO.Con.300° = 20±6  IKO.Con.240° = 20±5  IKO.Con.180° = 22±5  IKO.Con.60° = 24±5  IKO.Ecc.60° = 30±8  IKO.Ecc.120° = 26±6  IKO.Ecc.180° = 26±4  IKO.Ecc.240° = 28±5  Females:  ISO = 15±5  IKO.Con.300° = 11±2  IKO.Con.240° = 11±3  IKO.Con.180° = 12±3  IKO.Con.60° = 14±4  IKO.Ecc.60° = 18±4  IKO.Ecc.120° = 18±6  IKO.Ecc.180° = 18±3  IKO.Ecc.240° = 19±3 | ISO = 1.5  IKO.Con.300° = 1.5  IKO.Con.240° = 1.8 IKO.Con.180° = 2 IKO.Con.60° = 2  IKO.Ecc.60° = 1.5  IKO.Ecc.120° = 1.33  IKO.Ecc.180° = 2 IKO.Ecc.240° = 1.4 |
| Smith, et al., 2001 | Isometric; Isokinetic:  90°/s | Nm | Males:  ISO = 36.2±7.6  IKO = 30.2±5.2  Females:  ISO = 17.5±3.2  IKO = 14.1±2.0 | ISO = 2.46  IKO = 3.09 |
| Kramer and Ng, 1996 | Isometric; Isokinetic:  0°/s  60°/s  120°/s | Nm | Males:  ISO = 42±11  IKO.Con.60°/s = 37±11  IKO.Con.120°/s = 33±11  IKO.Ecc.60°/s = 41±10  IKO.Ecc.120°/s = 41±10  Females:  ISO = 22±7  IKO.Con.60°/s = 18±7  IKO.Con.120°/s = 15±6  IKO.Ecc.60°/s = 22±8  IKO.Ecc.120°/s = 22±7 | ISO = 1.82  IKO.Con.60°/s = 1.73  IKO.Con.120°/s = 1.64  IKO.Ecc.60°/s = 1.90  IKO.Ecc.120°/s = 1.90 |
